# Significance of PD1 Alternative Splicing in Celiac Disease as a Novel Source for Diagnostic and Therapeutic Target

**DOI:** 10.3389/fimmu.2021.678400

**Published:** 2021-06-16

**Authors:** Candelaria Ponce de León, Pedro Lorite, Miguel Ángel López-Casado, Francisco Barro, Teresa Palomeque, María Isabel Torres

**Affiliations:** ^1^ Department of Experimental Biology, Faculty of Health Sciences, University of Jaén, Jaén, Spain; ^2^ Department of Pediatric Gastroenterology, Virgen de las Nieves Hospital, Granada, Spain; ^3^ Department of Plant Genetic Improvement, Institute for Sustainable Agriculture, Spanish National Research Council (CSIC), Córdoba, Spain

**Keywords:** PD1/PDL, celiac disease, alternative splicing, gluten peptides, immune checkpoint

## Abstract

**Background:**

We have focused on the alteration of the PD-1/PD-L1 pathway in celiac disease and discussed the roles of the PD1 pathway in regulating the immune response. We explored the idea that the altered mRNA splicing process in key regulatory proteins could represent a novel source to identify diagnostic, prognostic, and therapeutic targets in celiac disease.

**Methods:**

We characterized the PD1 mRNA variants’ profile in CD patients and in response to gluten peptides’ incubation after *in vitro* experiments. Total RNA from whole blood was isolated, and the coding region of the human *PD-1* mRNA was amplified by cDNA PCR.

**Results:**

PCR amplification of the human PD-1 coding sequence revealed an association between the over-expression of the sPD-1 protein and the PD-1Δex3 transcript in celiac disease. Thus, we have found three novel alternative spliced isoforms, two of which result in a truncated protein and the other isoform with a loss of 14 aa of exon 2 and complete exon 3 (Δ3) which could encode a new soluble form of PD1 (sPD-1).

**Conclusions:**

Our study provides evidence that dietary gluten can modulate processes required for cell homeostasis through the splicing of pre-mRNAs encoding key regulatory proteins, which represents an adaptive mechanism in response to different nutritional conditions.

## Introduction

Alternative splicing is the process whereby the cell obtains different proteins from a single gene. Deregulations of the alternative splicing process are associated with the appearance of various aberrant splicing isoforms that may have pathological potential (1). Changes in the splicing pattern can lead to obtaining or losing a domain that could be critical in protein formation, resulting in an alteration in protein stability and/or subcellular localization (2). The isoforms generated from splicing can have similar, different, and even antagonistic functions, depending on the extent of the changes in the open reading frame, which involves the regulation of gene function. Alternative splicing events play critical roles in immune responses and have been documented in the immune system (3).

Celiac disease (CD) can be classified as an autoimmune disease, influenced by genetic, environmental, and immunological factors (4, 5). It is characterized by an abnormal immune response to prolamins from wheat and other cereals, such as rye and barley, which affects genetically susceptible people. The classic form of CD is manifested by an intestinal injury with villous atrophy, crypt hyperplasia, and inflammatory cell infiltrate (6, 7). Gluten peptides with immunogenic characteristics can trigger both an innate and an adaptive immune response, leading to the clinical and histological manifestations of CD (8). In the pathogenesis of CD, the CD4+ T cells of the lamina propria are the central element, which recognize gliadin peptides modified by the enzyme transglutaminase 2 (TG2), with HLA-DQ2/DQ8 restriction, releasing cytokines involved in inflammation and the development of histological disorders (9, 10).

Gluten is the main compound of wheat proteins and is rich in proline and glutamine residues, which makes it resistant to digestion at the gastrointestinal level. The partial digestion of gluten produces small peptides that cause autoimmune disease in celiac patients (11). Gliadins constitute the most immunogenic fraction of gluten, containing the main stimulating epitopes of T cells DQ2.5-glia-1 (PF/YPQPQLPY), DQ2.5-glia-2 (PQPQLPYPQ), and DQ2.5-glia- 3 (FRPQQPYPQ) (12, 13). The main epitopes are present on the 33-mer peptide, which is the main contributor to gluten immunogenicity (14). Gluten proteins’ immunogenicity is the result of canonical epitopes and their variants, so natural substitutions of canonical epitopes may contribute to wheat toxicity, with some being more abundant than the canonical epitopes (14). Gluten proteins have different immunogenic potential and are found in variable proportions among cereal species. For these reasons, it should be considered that amino acid substitutions in the variants of these epitopes can increase, reduce, or suppress CD response (14).

In previous studies, our research group used RNA interference technology (RNAi) to reduce the expression of specific gliadin fractions and immunogenicity with down-regulation of immunodominant peptides (15). Silencing of specific prolamin fractions by RNAi resulted in significant differences in the peptide composition and the number of peptides per protein identified in the PT-digested flour (16).

Programmed cell death-1 (PD-1) is a molecule member of the CD28 family that is expressed in T, B, and myeloid cells and, when activated, is released into blood plasma as soluble PD1 (sPD1) form (17). PD-1 has two ligands, PD-L1 (B7-H1) and PD-L2 (B7-DC), which are expressed in a variety of cells (18, 19). Interaction between PD-1 and its ligands results in a diverse range of pathological effects in T-cell activation, T-cell tolerance, and immune-mediated tissue damage. The axis PD-1 and its ligands have already been observed with an important role in autoimmune disease regulation (20).

Nielsen et al. (21) reported the presence of five splicing isoforms, including an exon 3-skipped PD-1(PD-1Δ3). Exon 1 encodes a short signal sequence; exon 2 encodes an Ig domain; exon 3 is made up of the stalk and transmembrane domains; exon 4 encodes a sequence of 12 aa that marks the beginning of the cytoplasmic domain; and exon 5 contains the C-terminal intracellular residues and an untranslated region (21). The PD-1Δ3 isoform, with the loss of the exon 3 that encodes the membrane domain, is secreted as a soluble form; whose function will be different, or even antagonistic, to membrane form (21). The presence of soluble forms increases the diversity and complexity of the PD-1/PDL pathway, because soluble forms can represent contributory factors to immune homeostasis and can mediate immunological diseases (22). The modulation of inhibitory and stimulatory pathways may represent the best strategy for treating autoimmune diseases (20).

Only few studies report the role of PD-1/PD-L1 in intestinal inflammation (23, 24). PD-1 is highly expressed on T cells in inflamed colon from IBD patients, and blockade of PD-L1 suppressed experimental colitis (14). The role of PD-L1 in gut mucosa tolerance is known, engagement of PD-1 on T cells by PD-L1 inhibits the activation and proliferation of effector T cells, inducing production of IFN-gamma and IL17A cytokines. PD-L1 expression is normally upregulated during inflammation to prevent overt tissue damage (25).

Previously, we focused on the alteration of the PD-1/PD-L1 pathway in celiac disease and discussed the role of the PD1 pathway in regulating immune response in celiac disease autoimmunity. Increased levels of soluble PD1 are found in patients with celiac disease, when compared to healthy controls, while the sPD1 level is significantly higher than the expression of the PD1 membrane isoform in CD patients (26).

The *PDCD1* gene has been a candidate gene in polymorphism studies (27, 28). Several single‐nucleotide polymorphisms (SNPs) have been identified: such as, PD1.1 (rs36084323), PD1.3 (rs11568821), PD1.5 (rs2227982), and PD1.9 (rs2227981), which affect the inhibitory functions of the PD‐1 receptor (29). An important function of PD-1 in maintaining the peripheral self-tolerance and prevention of autoimmunity is further supported by the reported association between single nucleotide polymorphisms (SNPs) in the human PD-1 gene with susceptibility to systemic lupus erythematosus (SLE) (27, 28) and rheumatoid arthritis (27, 29). It is not yet clear if these SNPs in *PDCD1*gene are a cause of or simply additional to PD-1 function.

One of the objectives of this study was to investigate the role that PD1 and its ligands have in regulating autoimmunity in celiac disease. Up to now, PD1 variants expression in human leucocyte populations has not been explored in celiac disease; it remains possible that expression pattern of alternative PD1 molecules in persons with CD contributes to its dysregulation as this protein is key in inflammation and autoinmune response. To explore this issue in this study, we characterized the PD1 mRNA variants’ profile in CD in response to gluten peptide incubation for *in vitro* experiments. We hypothesized that the presence of a particular SNP genotype may destabilize the PD1 pre-mRNA as it occurs in other human diseases. The genetic association study was conducted to investigate the possible associations between PD1 single nucleotide polymorphisms (SNPs) and celiac disease in a Spanish population. In summary, we have analyzed the relationships between dietary factor (gluten), PD-1/PD-L axis, genetic polymorphism of PD1 gene and the functions in celiac disease, finding differences in reactivity of individuals to gluten peptides.

## Experimental Section

### Ethics

The study protocol was approved by the local Ethics Committee of the Virgen de las Nieves Hospital (Granada, Spain) and carried out in accordance with the Helsinki Declaration. Written consent was obtained from the parents or legal guardians of the children involved for the publication of any potentially identifiable images or data included in this article.

### Patients

This study included patients with active celiac disease with gluten intake (n = 25) (**Table 1**) and healthy controls (n = 5, 60% female, 40% male, age range 7–12 years). The diagnosis of CD was determined by serological testing using anti-endomysial antibodies (AAEMs) and anti-tissue transglutaminase antibodies (AATGs), by typing of the CD-specific human leukocyte antigen (HLA), with confirmation of the disease by small intestine biopsy according to the Marsh classification (30).

**Table 1:** Clinical data of celiac patients.

Patient	Weight (kg)	High (Cm)	Atrophy grade	AAG	AEMA	ATGA	HLADQB1	HLADR
Age Range: Babies 0–2 years (56%)								
Celiac 1	9.70	80	4	30.8	1/160	28	0303,0601	7,15(2)
Celiac 2	9.67	79.5	4	68.4	1/160	38	0201,0202	3,7
Celiac 3	7.8	75	3	12	1/160	38	0302,0301	4,4
Celiac 4	9.7	74	4	199	1/160	18	0201,0202	3,7
Celiac 5	7.40	68.5	4	200	1/320	120	0201,0202	3,7
Celiac 6	13	89	4	30	1/160	28	0201/0101	3,7
Celiac 7	11.5	80.5	1	7.55	1/160	20	0201/0202	3,11
Celiac 8	8	76.5	4	200	1/320	90	0201/0202	3,7
Celiac 9	11.3	90	4	111	1/320	118	0201/0202	3,7
Celiac 10	14	92	4	49	1/5	70	0301/0202	11(5),7
Celiac 11	6.6	65	4	2.23	1/5	20	0502/0604	13,15(2)
Celiac 12	12.8	89.5	4	36	1/360	95	0201/0301	7,11
Celiac 13	11.5	83	1	55	1/320	90	0301/0501	11,10
Celiac 14	7.50	66	4	60	1/320	27.70	0201,0501	1,3
Children 3–12 years (44%)								
Celiac 15	13	95	3	65	1/80	25	0302/0301	4,4
Celiac 16	12.30	88	4	200	1/320	90	0201,0501	1,3
Celiac 17	21.5	118	4	19.2	1/160	28	0201,0301	3,11(5)
Celiac 18	26.5	128	4	102	1/320	97	0202,0301	7,11(5)
Celiac 19	14.3	100.4	3	31.5	1/160	28	0301,0302	4,11
Celiac 20	26	130	4	23.5	1/320	165	0202,0301	7,11(5)
Celiac 21	27	125	3	100	1/320	111	0201/0202	3,7
Celiac 22	11.5	89.5	4	144	1/5	10	0502/0602	15,16
Celiac 23	10.6	79	4	20.1	1/5	50	0201/0503	3,14(6)
Celiac 24	23	126	4	19.3	1/20	26	0201/0303	4,13(6)
Celiac 25	12.3	88	4	200	1/320	90	0201,0501	1,3

AAG, anti-gliadin antibody expressed as mg/L; AAEM, anti-endomysial antibody; AATG, anti-transglutaminase antibody expressed U/ml.

### Peripheral Blood Mononuclear Cell Isolation and Stimulation

PBMCs were isolated from 6 ml of heparinized blood by Histopaque gradient centrifugation (Sigma Aldrich, Saint Louis, MO, USA) and cultured at a density of 1 × 10^6^ cells/ml in RPMI-1640 culture medium (GIBCO, Grand Island, NY, USA Gibco, Thermo Scientific, Madrid, Spain) supplemented with 10% fetal bovine serum (GIBCO), 1% penicillin–streptomycin, and 0.1% gentamicin (Sigma Aldrich).

Gluten peptides were obtained by using combinations of different RNAi fragments designed to target d *γ*-gliadin fraction and glutenins. The RNAi lines and transformation vectors used were previously reported (31). Lines were obtained by using combinations of different RNAi fragments designed to target different gliadin fractions—the *ω*-, *α*- and *γ*-gliadin. After silencing by RNAi, there is a variation in the number of peptides per protein and its composition. In our study, we used the 33-mer peptide, as it is the main immunodominant toxic peptide for celiac patients, the wild-type line BW208 (BW), and the D623 line, as this has a higher number of peptides per protein than the wild-type line BW208, and shows an increase in *α*-gliadin peptides. After 48 h, the PBMCs were incubated with 10 µg/ml PHA (GIBCO) (positive T cell stimulation) for 24 h, and peptides (50 µg/ml) of cell medium were added in each well to stimulate lymphocytes. Supernatants were collected and stored at −80°C for later analysis.

### IFN-*γ* Production

The PT-digested protein extracts were used to study immunogenic potential by assaying IFN-*γ* release for *in vitro* assays. The supernatants from PBMC culture were collected after 24 h of peptide incubation and stored at −80°C for IFN-*γ* determination using a commercial ELISA kit in accordance with the manufacturer’s instructions (Thermo Fischer Scientific, Waltham, MA, USA Thermo Scientific, Madrid, Spain). The sensitivity of the assay was <2 pg/ml.

### Cell Proliferation Analysis

The BrdU Cell Proliferation Assay Kit (Millipore Chemicon, Temecula, CA, USA) for the *in vitro* quantitative detection of newly synthesized DNA from actively proliferating cells was used in PBMC cultures after 48 h of incubation with the different types of peptides. The stimulation index (SI) was calculated by dividing the mean absorbance by 10 at a wavelength of 450 nm after stimulation by the mean absorbance of T cells exposed to the culture medium alone (negative control), then divided by 10. The proliferation of PBMCs was expressed as the mean fluorescence intensity.

### RNA Isolation and cDNA Synthesis

Total RNA from whole blood was isolated using QIAamp RNA Blood Mini kit (Qiagen, Hilden, Germany) according to the manufacturer’s instructions. The quantity and quality of isolated RNA was measured using a NanoDrop Lite spectrophotometer (Thermo Scientific, Madison, WI, USA). Total RNA was converted into single-strand cDNA using a Maxima first-strand cDNA synthesis kit (Thermo Fisher Scientific). The quality of the cDNA was confirmed by PCR using human *β-actin* gene specific primers: Actin-F (5′-ATCTGGCACCACACCTTCTAC AATGAGCTGCG) and Actin-R (5′-CGTCATACTCCTGCTTGCTGATCCACATCTGC).

### PD-1 PCR Amplification, Fragment Cloning, and Sequencing

Amplification of the coding region of the human *PD-1* mRNA was performed by cDNA PCR using the primers PD-1-F (5′-GCGGCCAGGATGGTTCTTA) and PD-1-R (5′-TACTCCGTCTGCTCAGGGA) (**Supplementary Material**). The PCR program consisted of an initial denaturalization step at 94°C for 5 min and 40 cycles with 1 min at 94°C, 1 min at 65°C, and 1 min at 72°C, with a final extension of 5 min at 72°C. The amplified fragments were analyzed by electrophoresis in 2% agarose gels, eluted, and cloned into the pGEM-T Easy vector (Promega Corporation, Madison, WI, USA). Recombinant plasmids were sequenced on both strands by the dideoxy sequencing method.

### Genotyping the PD-1.3 and PD-1.5 Polymorphisms of the PD1 Gene

Two single nucleotide polymorphisms (SNPs) were analyzed in CD patients (n = 76) and healthy controls (n = 59) by the PCR-based RFLP restriction fragment length polymorphism method (**Table 2**). The first, (rs11568821), called PD-1.3, is located within intron 4 of the gene. The second, (rs2227981), called PD-1.5, is located within exon 5 of the gene.

**Table 2:** Analysis of the PD1 SNP polymorphisms by PCR-RFLP.

SNP	PCR primers^1^	Restriction endonuclease	Generated fragments
PD1.3 G/A (rs11568821)	PD1.3-F CCAGGCAGCAACCTCAAT	*Pst*I	G, 331 bp
	PD1.3-R GTCCCCCTCTGAAATGTCC		A, 276 bp, 55 bp
PD1.5 C/T (rs2227981)	PD1.5-F AGACGGAGTATGCCACCATT	*Alu*I	C, 249 bp, 84 bp
	PD1.5-R CACTGTGGGCATTGAGACAT		T, 180 bp, 69 bp, 84 bp

^1^Primers for PD1.3 were taken from Hoffmann et al. (32) and for PD1.5 from Ferreiros-Vidal et al. (33).

**Figure 1:**
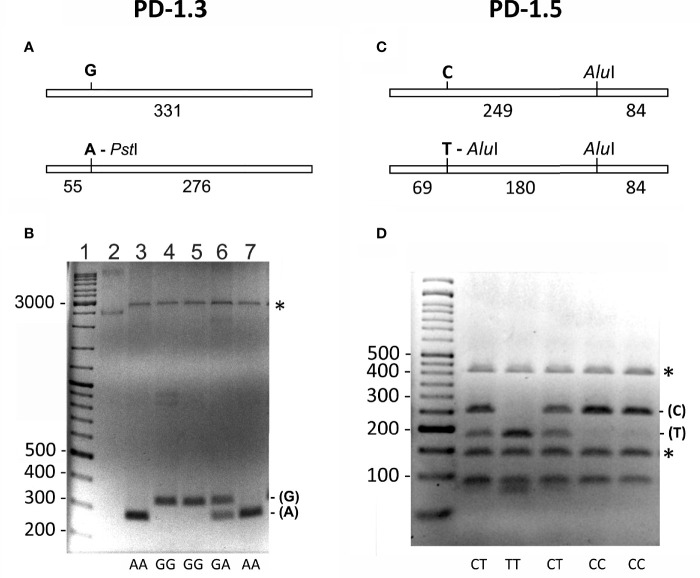
**(A, B)** Genotyping of the (G/A) PD-1.3 polymorphism of the PD1 gene. **(A)** Schematic representation of the 331 bp PCR amplified fragment containing the SNP. The presence of A in the SNP generates a target for the restriction endonuclease *Pst*I that generates two fragments of 55 and 276 bp. **(B)** Electrophoresis of the *Pst*I digested PCR products on a 2% agarose gel, showing the different banding patterns obtained for each genotype (AA, GG and GA). Lane 1 = DNA size marker. Lane 2 = undigested plasmid used as an internal control of the *Pst*I digestion. The numbers on the left indicate the size of DNA fragments in bp. Asterisk marks linearized form of the plasmid used as internal control for the *Pst*I digestion. **(C, D)** Genotyping of the (C/T) PD-1.5 polymorphism of the PD1 gene. **(C)** Schematic representation of the 333 bp PCR amplified fragment containing the SNP. The presence of T in the SNP generates an additional target for the restriction endonuclease *Alu*I that generates three fragments of 69, 180 and 84 bp. **(D)** Electrophoresis of the *Alu*I digested PCR products on a 3% agarose gel, showing the different banding patterns obtained for each genotype (CC, CT and TT). First lane is the DNA size marker. The numbers on the left indicate the size of DNA fragments in bp. Asterisk marks the two fragments generated by the DNA used as an internal control for the *Alu*I digestion.

Genotyping of the PD-1.3 was analyzed following Hoffmann et al. (32). A 331 bp containing the SNP was amplified by PCR using the primers PD1.3-F and PD1.3-R (**Figure 1A**). The PCR reactions were performed using the DreamTaq Green PCR Master Mix 2× (Thermo Fischer Scientific) using 50 ng of genomic DNA and 10 pmol of each primer in a 25 µl mixture. The PCR cycling profile was as follows: initial denaturation at 95°C (4 min), 35 cycles at 95°C (30 s), 60°C (1 min), 72°C (30 s), with a final elongation step of 72°C for 5 min. Aliquots of 5 μl of the PCR reaction mixture were analyzed by electrophoresis in 2% agarose gels to test the DNA amplification. Aliquots of 10 μl of the PCR reaction mixture were digested by adding five units of the restriction endonuclease *Pst*I (Sigma Aldrich) in a 40 μl reaction mixture that also contained 50 ng of the pUC19 plasmid. This plasmid was used as an internal control in the PCR product digestion. Only digestions with totally linearized plasmid were considered (**Figure 1B**).

For genotyping the PD-1.5 polymorphism, a 333 bp fragment containing the SNP was amplified by PCR using the primers PD1.5-F and PD1.5-R (**Figure 1C**). The PCR reactions were performed using the same conditions as for the PD-1.3 polymorphism. Aliquots of 10 μl of the PCR reaction mixture were digested by adding five units of *Alu*I (Sigma Aldrich, Saint Louis, MO) in a 40 μl reaction mixture. Then 50 ng fragment of the 16S rRNA gene of the ant *Messor structor* was added as an internal control for the digestion (34). When digested, this fragment produced two bands of 403 and 135 bp (**Figure 1D**). Only digestions where both bands were visible were considered.

### PD1/PDL1 and PDL2 Immunohistochemistry

Formalin-fixed paraffin-embedded biopsies from patients with active CD and control subjects were analyzed. About 4 µm thick sections were cut, deparaffinized, and rehydrated. Sections were microwaved in 10 mM of citrate buffer (pH 6.0) for antigen retrieval and cooled in phosphate-buffered saline. Endogenous peroxidase activity was quenched with 3% hydrogen peroxide. Sections were then treated with the protein-blocking agent, incubated with the primary antibody, followed by the biotinylated secondary antibody and the Streptavidin–Biotin Universal detection system (Immunotech, Marseilles, France). We used anti-human PD1, anti-human PDL1, and anti-human PDL2 antibodies (Abcam, Cambridge, UK). Negative control experiments were performed by incubating sections with isotype-matched IgG1.

### ELISA Quantification of sPD1/sPDL1 and sPDL2

We determined the levels of soluble PD1, PDL1, and PDL2 in serum of active celiac and non-celiac patients using a commercial ELISA kit in accordance with the manufacturer’s instructions (Sigma-Aldrich) (Invitrogen, Carlsbad, CA, USA) (Thermofisher Scientific). For each assay, the standards as well as the samples were tested in duplicate. To estimate the cytokine concentration (pg ml^−1^). The sensitivity of the assays was <2 pg/ml. The concentrations of sPD1, sPDL1, and sPDL2 were determined from the optical density according to standard curves.

### Statistical Analysis

Statistical analysis of the data was done using Statgraphics software (StatPoint Technologies, Warrenton, VA, USA). The chi-square test was used for comparison of qualitative data. Quantitative data were expressed as a median, and the Mann–Whitney U test was used as a test of significance for comparing two groups. The SNPs were tested for Hardy–Weinberg equilibrium, then their genotypic and allelic disease association analysis was performed. For all tests, the results were considered statistically significant for p <0.05.

## Results

### Diagnosis: Serological, Genetic, and Histological Analysis

The endomysial antibody (AEMA) was positive in all CD patients included in this study. The anti-transglutaminase antibody (ATGA) was elevated (range 16 ≥ 200) in 95% of CD patients. There were no patients with IgA deficiency. All patients were active for celiac disease (**Table 1**).

Patients with CD had classic signs of the disease in intestinal biopsies with the presence of villi, with total or partial atrophy and increased lymphocyte infiltration. These histological alterations were classified according to the Marsh criteria (types I–IV). Among the celiac patients included in this study, 16% had Marsh III lesions (partial or complete atrophy of the villi and crypt hypertrophy), 76% had Marsh IV hypoplasia (totally atrophied villi, that is, completely flattened), and 8% had Marsh I lesions (increased intraepithelial lymphocytes, but without villus atrophy) (**Table 1**).

HLA genotype frequencies among these patients with CD were as follows: HLA-DQ2 (DQA1*0501, DQB1*0201) was the most frequent allele in the patients (76%). Three patients (24%) were carriers of DQ8 (DQA1*03:01,*03:03–DQB1*03:02).

### Stimulatory Response of PBMCs From Patients With CD

**Figure 2:**
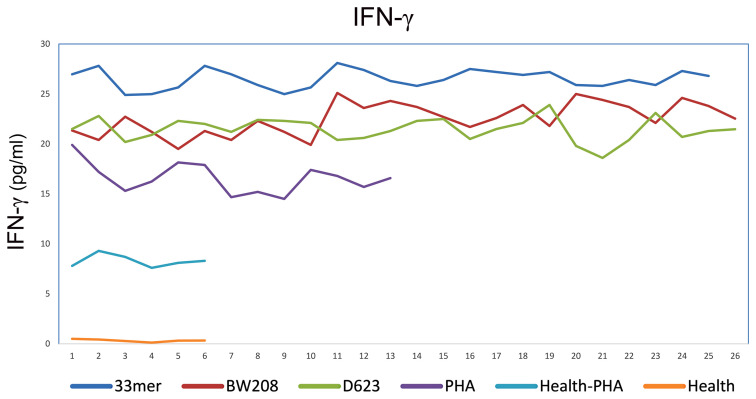
IFN**-**gamma release by PBMCs of CD patients stimulated with different peptides and PHA as positive control and of healthy controls stimulated and unstimulated with PHA. In axis X the patients and in axis Y the levels of IFN-gamma expressed in pg/ml.

The specific cell proliferation capacity and the amount of IFN-*γ* released against gluten peptides were studied to determine the ability to activate the stimulatory response of PBMC in patients with CD. The immunogenic potential of gluten peptides was quantified by the stimulation index in cultures of patients with CD subjected to a diet containing gluten. In addition, a control group was included without the addition of gluten peptides to the cell cultures with the same culture conditions; these were the reference values to compare with the effect of the peptides in cells of patients with CD. As a positive control assay, we stimulated T-cell by adding phytohemagglutinin (10 μg/ml PHA), and the production of IFN-*γ* was measured to reflect the proliferation function of T-cells. We chose to study the 33-mer peptide, BW208 and D623 wheat lines, due to their higher cell proliferation and IFN-*γ* values. The highest values of IFN-*γ* released were found in the supernatant of T-cells incubated with 33-mer (26.5 ± 1.6 pg/ml), BW208 (22.5 ± 2.6 pg/ml) and D623 wheat line (21.5 ± 1.3 pg/ml), respectively (**Figure 2**). The value of IFN-*γ* stimulated T-cell by PHA was 16.5 ± 1.8 pg/ml. In healthy controls, the levels of IFN-*γ* were 0.3 ± 0.4 pg/ml and 8.3 ± 0.7 pg/ml in unstimulated and PHA stimulated T cells, respectively (**Figure 2**).

The results of cell proliferation from celiac PBMCs clearly showed that the peptides 33-mer (SI = 34.7 ± 1.4), BW208 (SI  =  29.7 ± 1.5), and D623 (SI   = 24.6 ± 1.2) wheat lines induced a weak proliferative response in comparison with the negative control (SI   = 4.7 ± 0.4).

**Figure 3:**
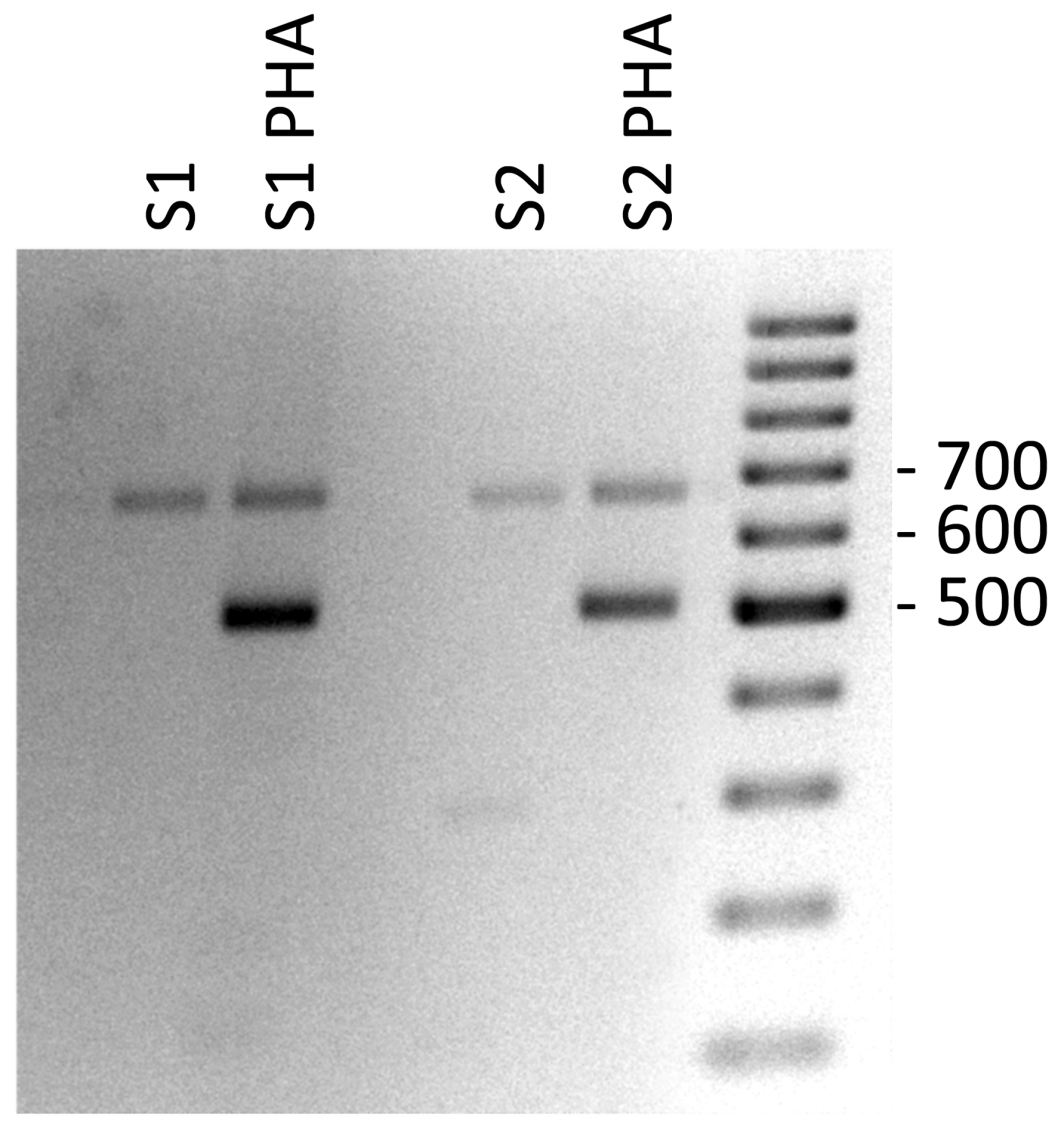
Gel electrophoresis of PD-1 cDNA amplification products from PHA stimulated PBMCs of celiac patients as control positive of T cell stimulation.

We also analyzed the PHA-associated expression of PD1 mRNAs of PBMCs by reverse transcription and subsequent polymerase chain reaction (RT-PCR) in celiac patients and by flow cytometry in healthy controls. In PBMCs without PHA stimulation, we found expression of the full PD1 variant (687 bp) representing the membrane-bound isoform (**Figure 3**). In PHA stimulated cells, an additional band was obtained. The sequencing of that fragment revealed it was an alternatively spliced transcript of the PD1, lacking exon 3. This PD-1 Dex3 variant encodes the soluble form of PD1 (sPD-1); see **Figure 3**.

**Figure 4:**
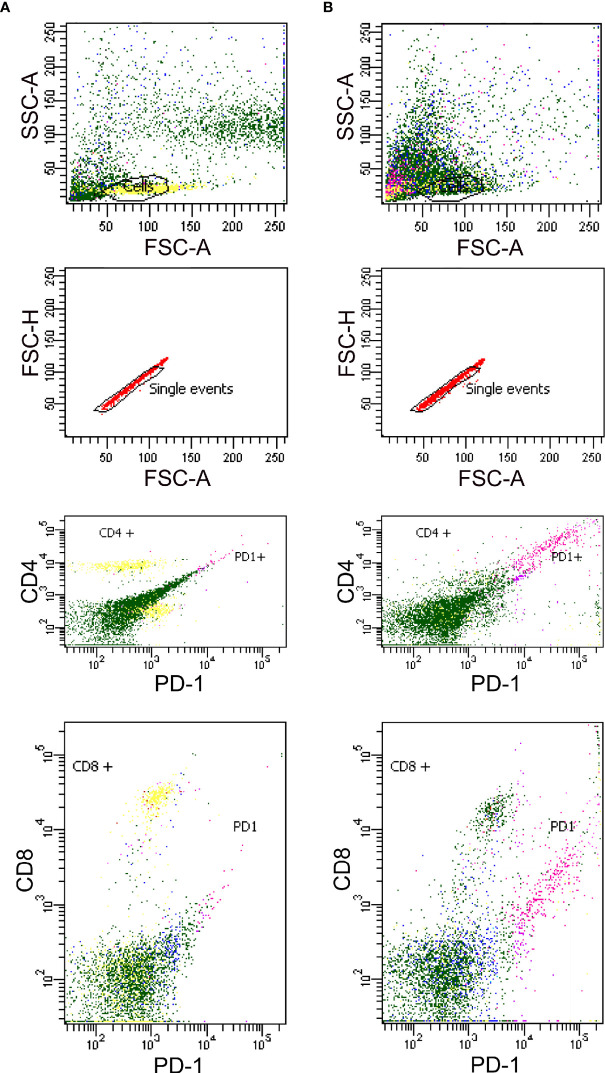
Detection of PD1 in Human PBMCs from healthy controls by flow cytometry either unstimulated **(A)** and stimulated with PHA **(B)**.

By flow cytometry were analyzed both CD4^+^ and CD8^+^ populations for detection of PD1 in healthy controls, either (A) untreated or (B) treated with PHA. As indicated in **Figure 4**, we found an increase in PD1 expression correlated with T-cell stimulation when PHA was added under the conditions of culture employed in this study. Generally, CD4^+^ populations demonstrated predictable increases in PD1 expression, while CD8^+^ populations were slightly less likely to follow this pattern, indicating that the cells were successfully stimulated (**Figure 4**).

### PD1/PDL1/PDL2 Expression in Celiac Disease by Immunohistochemistry

**Figure 5:**
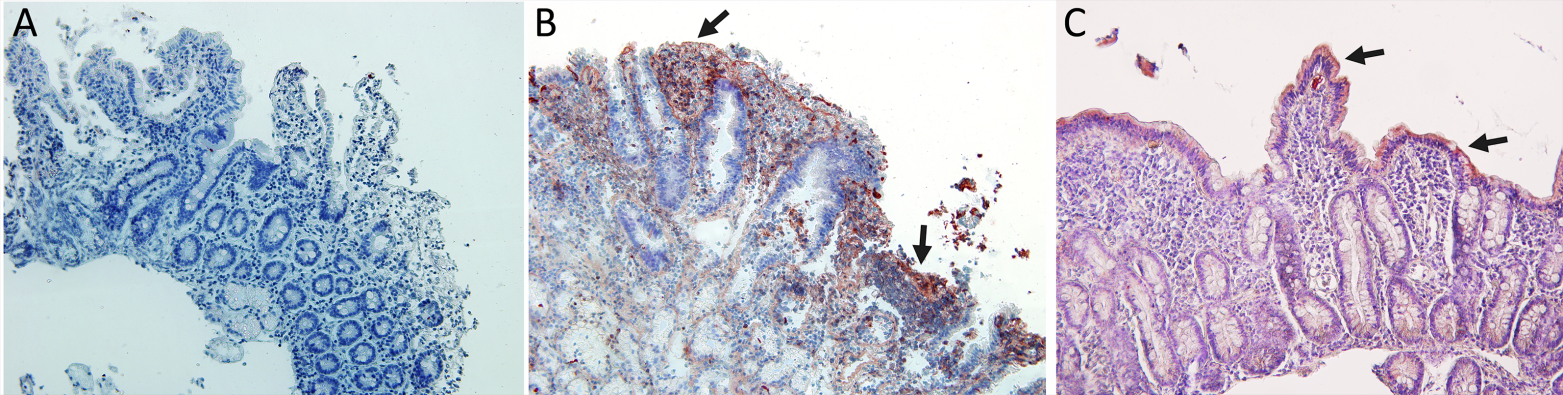
Immunohistochemistry analysis: **(A)** Negative immunostaining for PD1 in celiac disease patients. **(B)** Immunostaining for PDL1 in celiac disease patients showing expression in lamina propria cells and in Lieberkhün crypts. **(C)** Immunostaining for PDL2 in CD patients showing expression in epithelial cells. Magnification 200×. Arrows: cells with immunoreaction.

In this study, we tested two antibodies that recognized PD1 protein which different immunogens. One of them was a recombinant anti-PD1 antibody in which immunogen is a synthetic peptide within Human PD1 aa 1–100 (N terminal) with membrane cellular localization and contains the one Ig-like V-type (immunoglobulin-like) domain. The other was a monoclonal antibody in which immunogen is an NK-like leukemia cell line that expresses PD1, with membrane cellular localization, and also contains the one Ig-like V-type (immunoglobulin-like) domain. The epitope is in the range 24–170 aa protein. We found the same immunohistochemistry results with the two antibodies tested, giving negative PD1 expression in biopsies of patients with celiac disease (**Figure 5A**).

Indeed, all patients tested showed a positive expression of PDL1 and PDL2 with a different degree of immunoreactivity. PD-L1 is expressed on lamina propria cells of active CD patients (**Figure 5B**), and PDL2 is expressed on intestinal epithelial cells of active CD patients (**Figure 5C**).

### Detection of sPD1/PDL1 and sPDL2 in Serum From Patients With CD

**Figure 6:**
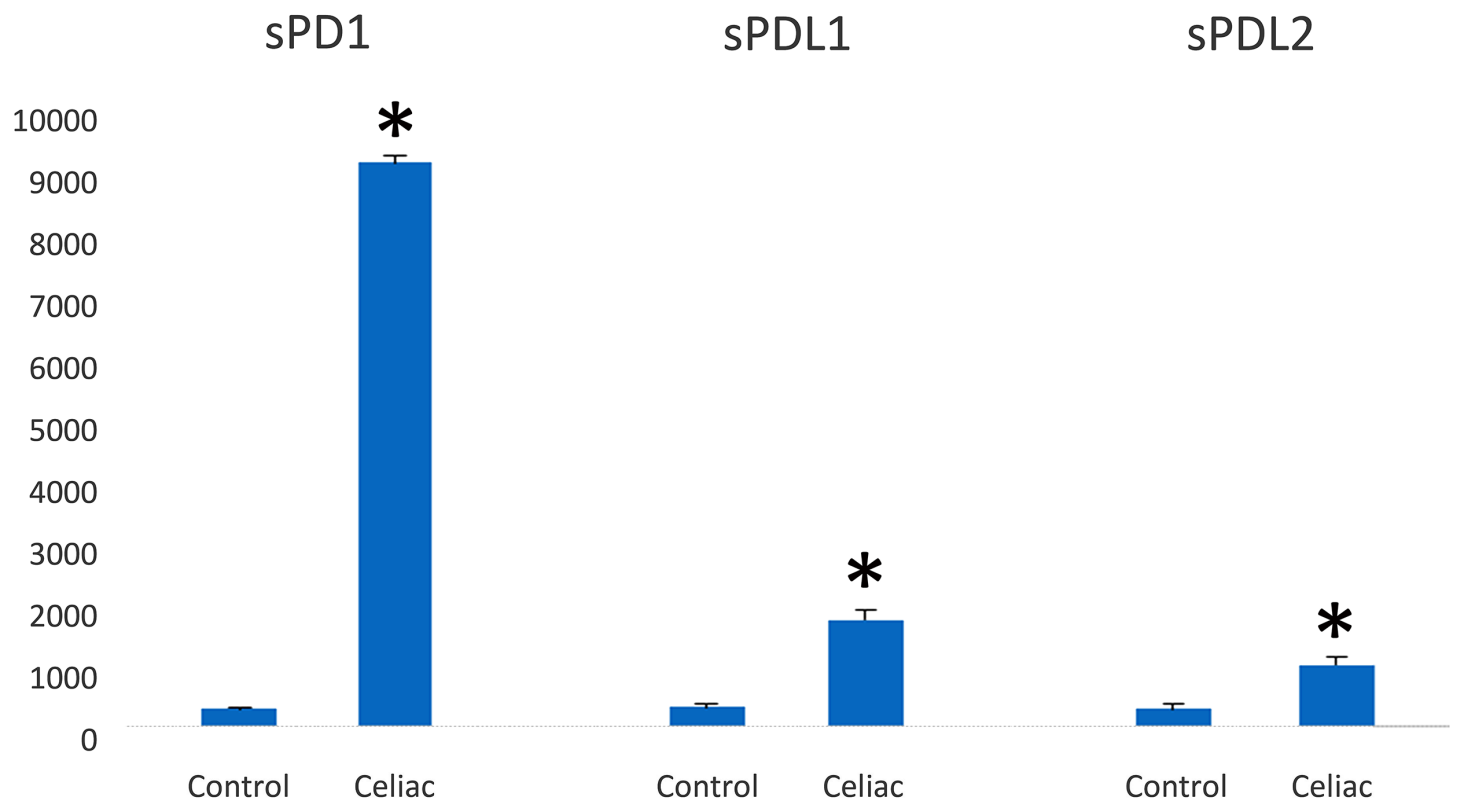
PD-1/PD-L1 expression in the serum of celiac disease patients and healthy controls (pg/ml). PD-1 and PD-L1 are highly expressed in serum from celiac disease patients (n = 25) in relation to healthy controls (n = 5). Significant difference at *p < 0.05 is shown.

ELISA was used to examine the serum level of sPD-L1 and sPD-L2 in patients with CD to test its utility as a candidate biomarker. We showed that levels of sPD-1 were considerably higher in the serum of patients with celiac disease (n = 25) compared with healthy controls (n = 5) (9,123 ± 120 pg/ml *vs* 288 ± 36 pg/ml); see **Figure 6**. The mean levels of PDL1 and PDL2 were 1,723 ± 150 pg ml^−1^ *vs* 320 ± 65 pg ml^−1^ and 997.10 ± 126 *vs* 320 ± 78 in sera from patients with CD and healthy control, respectively.

### Identification of Alternatively Spliced Variants of PD-1 mRNA in Peripheral Blood Mononuclear Cells Stimulated With Gliadin Peptides

In this study, we describe for the first time the expression of different splice variants for the PD-1 gene in CD patients. In an attempt to evaluate whether the regulation of splicing could be modified during the course of CD, we studied the pattern of PD1 isoforms in peripheral leucocytes from patients with CD, in comparison with healthy controls.

**Figure 7:**
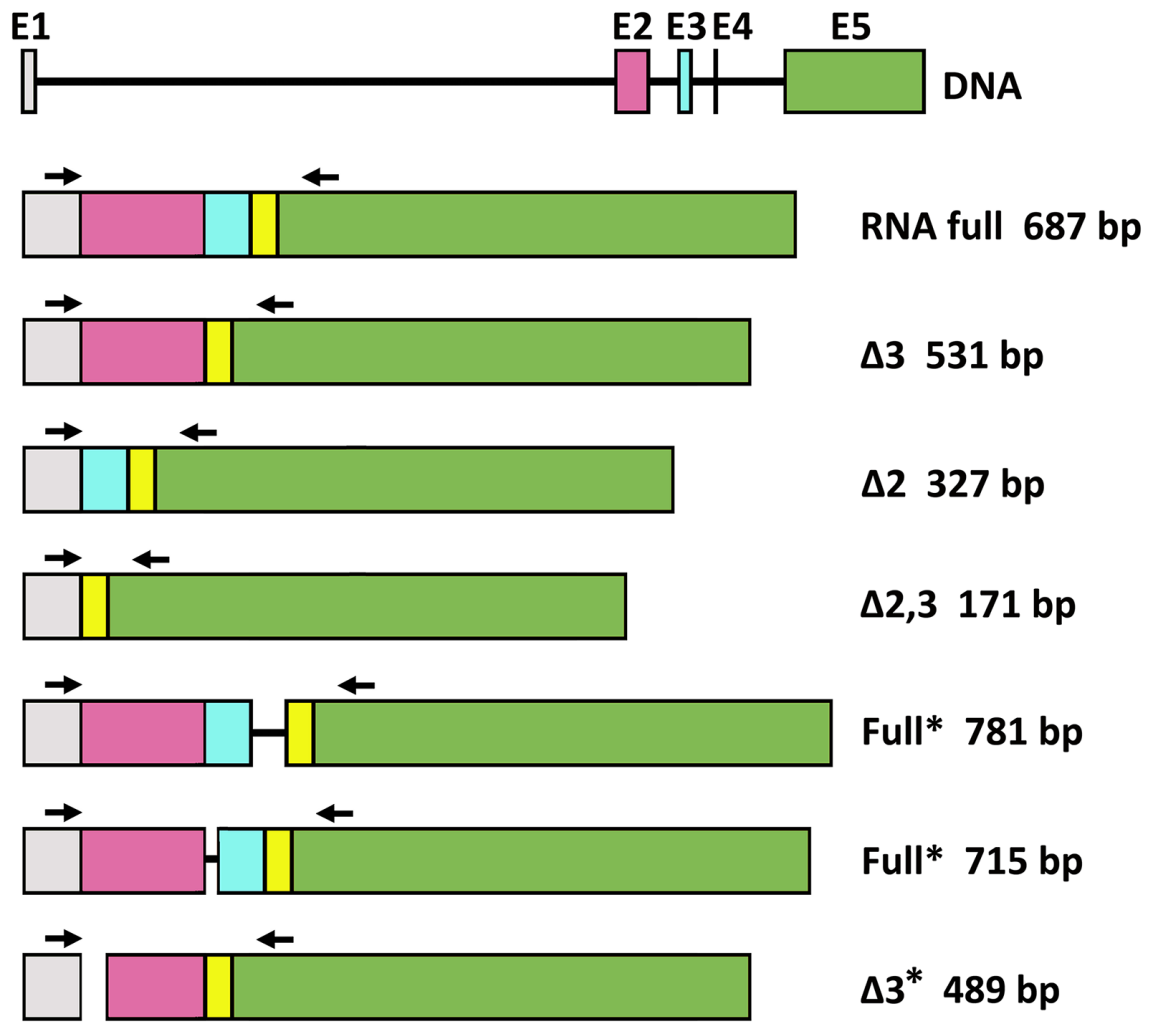
Schematic diagram for PD1 splicing variants found in this study. flPD-1, PD-1Dex3, PD-1Dex2, PD-1Dex2,3 with sizes about 687, 531, 327, and 171 bp, respectively. New alternative spliced isoforms that retained part of different introns, with sizes of 781 and 715 bp, and isoform with a loss of 42 bp from exon 2 and a complete exon 3 (Δ3) with a size of 489 bp. Asterisks indicate the new isoforms found in this study.

The effect of gluten peptide intervention on the expression pattern of splicing machinery components was evaluated in PBMCs from patients with celiac disease. PBMCs cultured from patients with CD and healthy controls were stimulated with different types of peptides (33-mer, BW, and D623). PCR amplification of the human PD-1 coding sequence revealed the expression by human PBMCs of four splice variants: flPD-1, PD-1Dex3, PD-1Dex2, PD-1Dex2,3 with sizes about 687, 531, 327, and 171 bp, respectively (**Figure 7**). We also found two new alternative spliced isoforms that retained part of different introns, with sizes of 781 and 715 bp, as well as another isoform with a loss of 42 bp from exon 2 and a complete exon 3 (Δ3) with a size of 489 bp (**Figure 7**). All amplified products were cloned and sequenced. The largest band (687 bp) represents the membrane-bound form, showing complete homology with the published membrane PD-1 sequence (GenBank Accession No. NM_005018). The 531, 327, and 171 bp correspond to previously described variants (PD-1Dex2, PD-1Dex3, and PD-1Dex2,3) that lack the exon 2, exon 3, or both (**Figure 7** and **Supplementary Figure S1**).

**Figure 8:**
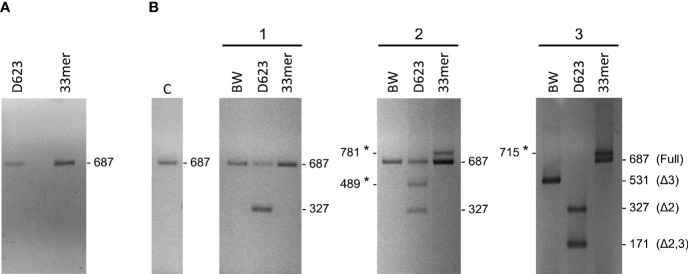
Gel electrophoresis of PD-1 cDNA amplification products from PBMCs stimulated with gluten peptides in healthy controls **(A)** and in celiac patients **(B)**. With the used primers in the present study, the sizes of the respective PCR products were 687 bp for the full-length PD-1 transcript, 531 bp for PD-1Dex3, 327 bp for PD-1Dex2, 171 bp for PD-1Dex2,3, and three new isoforms with 781, 715, and 489 bp. Asterisks indicate the new isoforms found in this study.

The PMBC stimulation with BW wild-type peptide showed the expression of the PD1 form (687 bp) representing the membrane-bound form while also producing an alternatively spliced transcript of the PD1 with the sequence encoded by exon 3 being skipped, encoding the soluble form of PD1 (**Figure 8**). The stimulation with the D623 line that presented a higher number of peptides per protein than the BW wild-type line showed expression by human PBMCs of four splice variants: flPD-1, PD-1Dex3, PD-1Dex2, PD-1Dex2,3, presenting variability of expression in each patient with celiac, as shown in **Figure 8**. In patient 2, the stimulation with line D623 presented a new PD1 isoform (Δ3*, band 489 bp) with a loss of 42 bp at the beginning of exon 2 and without exon 3 (**Figure 7** and **Supplementary Figure S1**). This deletion does not change the reading frame (**Supplementary Material S4**) and could produce a protein which lacks the first 14 aa codified by exon 2. This isoform could encode another soluble and new isoform of PD1 (sPD-1).

Stimulating the PMBCs with 33-mer showed the expression of two PD1 alternative spliced isoforms with sizes of 781 and 715 bp, respectively (**Figures 7**, **8B**, **Supplementary Figure S1**). In patient 2, a large 33m-2 band of 781 bp retains all exons and a 95 bp fragment of the end of the intron that separates exons 3 and 4 (Splicing 3′ site that establishes the end of one intron and the beginning of the next exon). The 95 bp sequence introduces a premature stop codon (TAA) that could produce a truncated protein without the aa encoded by exons 4 and 5 (**Supplementary Figure S2**). In patient 3, a large 33m-3 band of 715 bp retains all exons and a 25 bp fragment at the beginning of the intron between exons 2 and 3, with the last three bp of this intron (Splicing 5′ site that establishes the end of one exon and the beginning of the next intron); see **Figures 7**, **8B** and **Supplementary Figure S1**. These nucleotides generate a change in the reading frame and therefore a change in the aa sequence from this point, so the protein will surely be non-functional (**Supplementary Figure S2**). It was not possible to establish a correlation between the degree of inflammatory activity of celiac disease according to the Marsh classification and the expression of the different isoforms of PD1 described in this study. The control group (C), corresponding to PMBCs of patients with CD without posterior peptide stimulation *in vitro*, revealed the expression of the PD1 form (687 bp), representing the membrane-bound form, while other CD patients present expression of the soluble form of PD1 (sPD-1); see **Figure 8B**.

Finally, gel electrophoresis of PD-1 cDNA amplification products from PBMCs stimulated with gluten peptides in healthy controls showed the expression of the PD1 form (687 bp), representing the membrane-bound form (**Figure 8A**).

### PD‐1 Genotype and Allele Frequencies

The allele and genotype frequencies of PD-1.3 and PD-1.5 SNPs are shown in **Table 3**. In this study, the observed genotype frequencies in the control and CD groups were consistent with the Hardy–Weinberg equilibrium.

**Table 3:** Allele and genotype frequencies of polymorphic sites of the PD1 gene in celiac Spanish population.

PD-1.5 SNP frequencies			PD-1.3 SNP frequencies		
Genotypes	Control	Celiac	Genotypes	Control	Celiac
CC	42% (24)	43.5% (33)	GG	65% (37)	67% (51)
CT	49% (28)	46% (35)	GA	31.5% (18)	29% (22)
TT	12% (7)	10.5% (8)	AA	3.5% (2)	4% (3)
Alleles	Control	Celiac	Alleles	Control	Celiac
C	64.4% (76)	66% (101)	G	80.7% (92)	81.6% (124)
T	35.6% (42)	33.5% (51)	A	19.3% (22)	18.4% (28)

For the PD-1.3 SNPs, the frequency of the GG genotype was higher in patients with CD as well as in healthy controls, while the AA genotype was less abundant in both groups. Analysis by Pearson’s х2 and Fisher’s exact test showed there was no significant association between CD and the genotypes and alleles (p>0.05). Moreover, there was no meaningful association between the gender of patients with CD and specific PD-1.3 genotypes. In the CD group, the odds of celiac for AA were 1.08 times as great as the odds of celiac for GG, and the odds for AG were 0.89 times as great as the odds for GG. Meanwhile, the healthy control group odds for AG were 1.13 times as great as the healthy control group odds for GG, and for AA this was 0.92 times as great as the odds for GG. However, this difference in CD susceptibility between the AA and GG genotypes was not statistically significant.

For the PD-1.5 SNP, the frequency of the CT genotype was higher in patients with CD and in healthy controls, while the TT genotype was less abundant in both healthy control groups. Analysis by Pearson’s х2 and Fisher’s exact test showed there was no significant association between CD and the genotypes and alleles (p>0.05). Moreover, there was no meaningful association between the gender of patients with CD, while CC and TT genotypes were the most and least frequent genotype in the CD and healthy control.

Susceptibility to CD among subjects with the TT genotype seemed much higher than that among the subjects with the CC genotype; the odds ratios of CD patients for TT was 0.83 times as great as the odds of celiac for CC. In contrast, the odds ratios of healthy control for TT was 1.20 times as great as the odds ratios of the healthy control for CC. However, this difference in CD susceptibility between the TT and CC genotypes was not statistically significant.

Nevertheless, a small difference between the female and male patients could not be excluded completely due to the small number of male patients with CD in this study.

## Discussion

Celiac disease is a chronic and systemic autoimmune pathology characterized by a reaction to gluten in genetically predisposed individuals. It causes severe damage to the mucosa of the small intestine, with atrophy of the intestinal villi causing poor absorption of nutrients (35). The disease is polygenic involving genes from the major histocompatibility antigen (MHC) complex such as HLA DQ2 and DQ8 and, less frequently, other non-MHC genes (35). Studies of genome-wide association scans have demonstrated gene alterations at the level of regulators of the immune response in celiac disease (36, 37).

PD-1/PD-L1 binding prevents an excessive immune response and protects tissues from damage through the induction of immune tolerance in normal tissues (38). The inflammatory signaling and epigenetic alterations regulates the PD1/PD-L1 expression (39). In this study, we tested two antibodies that recognized PD1 protein with different immunogens: (i) a synthetic peptide within Human PD1 aa 1–100 (N terminal), and (ii) with the epitope in the range 24–170 aa protein. Both antibodies with membrane cellular localization contain one Ig-like V-type (immunoglobulin-like) domain. We demonstrated a negative expression of PD1 in the intestinal biopsy of active CD patients, with the same results for the two antibodies tested suggesting an important role for the PD-1/PDLs pathway in regulating mucosal tolerance *in situ*.

In a previous study, we assessed cell surface expression of PD1 on freshly isolated PBMCs by flow cytometry analysis. In CD patients, CD4+ and CD8+ T cells presented significantly decreased PD-1 protein expression (26). Thus, PD-1 function would be compromised in CD4+ and CD8+ T cells, indicating the establishment of an inappropriate activation state (40). A dysregulation of immunosuppression mechanisms that can lead to abnormal and persistent T-cell activation and cytokine production occurs in celiac disease. PD1 plays a crucial role in the tolerance mechanisms of central and peripheral T cells, helping to protect tissues from autoimmune responses. Without PD1 expression, excessive tissue damage mediated by the immune response can have devastating consequences in the protection of self-tissues from autoimmune responses.

The expression of sPD-1 could increase the maturation of dendritic cells (DCs), which could be accompanied by upregulation of major histocompatibility complex II (MHC II) (40). sPD-1-regulated DC maturation is mediated by activated T lymphocytes and may be influenced by increased T cell responses (40). Soluble PD-1 can play an adjuvant role in enhancing antigen-specific T-cell immunity responses. A deficit in PD1 gene expression has been shown to result in inadequate suppression of autoreactive lymphocytes, an aberrant presence of activated T cells and autoantibody production (40).

In this study, we found expression of PD-L1 and PD-L2 in serum and in intestinal biopsies of patients with active CD. These findings are consistent with other studies that show a higher expression of PD-L1 in other human autoimmune diseases. PD-L1 is known to be upregulated in inflamed intestinal tissue (41, 42). PD1 expressed on the surface of activated T cells, by binding to its PD-L1 ligand, sends inhibitory signals to T cells; this being a significant molecular mechanism for the control of antigen-specific T cells, thus avoiding excessive tissue damage induced by immune responses (43). In relation to cytokine expression, interferon (IFN)-*γ* plays an important role in the positive regulation of PD-L1 expression in tumor tissues (44).

In addition to the inhibitory effect of PD-L1 on inflammation, previous studies also showed that epithelial expression of PD-L1 induced by bacterial pathogens could inhibit T-cell functions (45). Thus, some species of commensal bacteria may contribute to chronic inflammation by upregulating the epithelial expression of PD-L1 in an inflammatory environment to inhibit the function of T cells in the mucosa.

PD-L2, as a receptor of PD1, is involved in the costimulatory signal essential for T-cell proliferation and IFN-*γ* production (46). PD-L2 generally expressed at a lower level may favor PD-L1 as the primary binding ligand of PD-1, except during Th2 responses when PD-L2 is upregulated (47, 48). Several research groups have shown that PD-L2 expression can be induced on a wide variety of other immune cells and non-immune cells depending on microenvironmental stimuli (49–51). PD-L1 and PD-L2 expression depends on distinct stimuli, and their expression patterns suggest both overlapping and differential roles in immune regulation (11).

Few studies have assessed PD-L2 expression in autoimmune diseases, especially in SLE (33, 52). We found PD-L2 expression on the intestinal biopsy from patients with CD. An interesting speculation would be that, in CD, the soluble PD-L2 could act as a decoy ligand to increase PD-1 activation of immune cells to further amplify the immunopathological damage. Additional studies would be required, however, to test this theory.

Wan and colleagues demonstrated that the soluble PD-1 in serum of RA patients was the translational product of the PD-1Δex3 mRNA transcript (53). We identified the over-expression of the sPD-1 protein and the PD-1Δex3 transcript in CD patients. These results provide new evidence that PD-1 is associated with CD and that its soluble form might play a key role during the phase of T cell exhaustion and the primary activation of T cells. Soluble PD-1 can be used as an adjuvant to increase T cell immunity. This study suggests that it is likely that the soluble PD-1Δ3 isoform still retains the ability to bind to PD-L1/PD-L2 and that, by interfering with its signaling pathway, it has antagonistic effects on PD-1. In patients with CD, the excessive sPD-1 could serve as an “antibody” to block the PD-1/PD-Ls pathway and lead to aberrant T-cell proliferation.

Soluble forms of many immune regulatory molecules, both co-stimulatory and co-inhibitory molecules, are detected in plasma of CD patients including sCTLA-4, sHLA-G, sCD27, and sIL-33 (54–56). These soluble forms are produced through alternative splice variants. sCTLA-4 is present in serum as a functional protein and correlates with mucosal injury (54)

We have identified four alternative spliced PD-1 mRNA transcripts of PD-1 (PD-1Dex2, PD-1Dex3, PD-1Dex2,3, and PD-1Dex2,3,4) in addition to the full length isoform encoded by exon 1 (leader peptide), exon 2 (extracellular IgV-like domain), exon 3 (transmembrane domain), exons 4 and 5 (intracellular domain). These transcripts were verified by cloning and sequencing, and the different splice variants did not represent amplified artifacts during PCR reactions.

The PD-1Dex2 mRNA transcript is generated from an alternative junction of the PD-1 gene where the sequence encoded by exon 2 is deleted. If this PD-1Dex2 mRNA transcript were translated, the protein product would be expressed as a molecule membrane lacking its binding properties to PD-L1/2 receptors. This splicing does not affect the reading frame and the biological significance of this mRNA transcription could be the result of a splicing error occurring in parallel with increased mRNA expression during cell proliferation development.

Similarly, we found expression of the PD-1Dex2,3 mRNA transcript in PBMC from patients with celiac disease subsequently stimulated with gluten-derived peptides. We have no evidence of any biological function of the putative protein encoded by this transcript, since this truncated protein would lack PD-L1/2 receptor binding properties. However, the PD-1Dex2,3 transcript does not affect the reading pattern and, if it were translated, it would introduce glycine at position 26 compared to aspartic acid in the complete form fl-PD1

Two new splice alternatives of PD1 variants that retain part of different introns with sizes of 781 and 715 bp were found. The 781 bp band has all the exons and a 95 bp sequence that introduces a premature stop codon (TAA) that leads to a loss in the region encoded by exons 4 and 5 as a result of a truncated protein. This protein with premature termination will be more or less serious depending on the area in which it occurs. The band at 715 bp has all the exons and a 28 bp fragment that corresponds to the beginning of the intron between exons 2 and 3. The reading pattern changes from the zone of the intron, and a totally different amino acid sequence is generated, so the protein will surely be non-functional.

Finally, a new isoform of 489 bp that has a deletion at the beginning of exon 2, being a multiple of three nucleotides, was found. This isoform does not change the reading pattern. The biological importance of this new mRNA transcript is unknown, although the level of this transcript was lower compared to the other transcripts we found in patients with celiac disease. This mRNA transcript would correspond to a new soluble isoform that would probably have antagonistic effects on the membrane shape by interfering with its signaling pathway and could also preserve the ability to bind to the PD-L1/PD-L2 ligands.

The PD-1 gene is located on chromosome 2q37.3 near another region 2q33.3 related to autoimmunity (57). To determine the associations between these PD-1 genetic variations and CD in the population of southern Spain, PD-1 SNPs were evaluated in genomic DNAs extracted from control groups and from patients with CD.

One of the selected polymorphisms was the PD-1.3 SNP, since it alters the inhibitory effect of PD-1 and increases the activity of lymphocytes. Substitution of guanine (G) for adenine (A) at nucleotide + 7,146 in intron 4 alters transcriptional regulation and PD-1 expression at this polymorphic site during disruption of runt-related transcription factor 1 binding (RUNX1) (28, 58). PD1.5 polymorphism does not exert any change in the final amino acid structure of the protein, and the link imbalance between the PD1.5 variation and other PDCD1 gene polymorphisms can lead to modify expression at the mRNA and protein level.

To our knowledge, this study primarily showed that PD1.3 and PD1.5 polymorphisms were not associated with risk of CD, possibly indicating the absence of any interaction between them. We have observed a tendency for an increase in PD1.3 G and PD1.5 C alleles in patients with CD in comparison with healthy controls, although this difference was not statistically significant. More such studies with a larger sample size would be needed in the Spanish population to confirm these observations. In addition, it must be considered that there may be other SNPs that we have not been able to identify in the current study.

Based on the results shown, we can suggest that the splicing machinery would act as a biological sensor to adapt gene expression to the pathophysiological conditions that arise (59). Dysregulation of the gene could lead to an imbalance in the splice variants present in cells at any given time and in response to external factors. Among these factors, the existence of gliadin-derived peptides in the serum of patients with CD could modulate the expression of relevant splice components and the function of the splicing machinery. Our study provides primary evidence that a dietary intervention of gluten peptides could alter the expression pattern of the splicing machinery at risk for CD. In this study, although we observed splicing complex formation in the presence of active peptides, it is not possible to use these assays to determine whether the active peptides inhibited splicing by interfering with conformational rearrangements in the splice.

The alternative splicing process may represent a physiological mechanism to maintain cell homeostasis. This is suggested in different studies that show that nutrients can modulate gene expression and, in particular, the splicing of pre-mRNA that encodes regulatory proteins (60). Minimal alterations in the alternative splicing process could lead to the production of deficient proteins that contribute to the development of some human diseases (61).

## Conclusions

In view of our results, we propose that gluten peptides may modulate the processes necessary for cellular homeostasis in a specific way through the alteration of gene expression; in particular in the splicing of pre-mRNA that encodes regulatory proteins’ key, as PD1. Our results indicate that alternative PD1 splicing could explain the complex pathophysiology that occurs in each patient with CD, and it can be hypothesized that there is differential regulation of PD1 splice variants in CD. These alterations in the production of different isoforms of PD1 could contribute to the development of CD.

Since PD1.3 and PD1.5 polymorphisms were present in both patients with CD and in healthy controls and no significant evidence was observed between both groups, it could not be concluded that this polymorphism was associated with CD.

## Data Availability Statement

The raw data supporting the conclusions of this article will be made available by the authors, without undue reservation.

## Ethics Statement

The local Ethics Committee of the Hospital “Virgen de las Nieves” (Granada, Spain) approved the study protocol. Written consent was obtained from parents or legal guardians of children involved.

## Author Contributions

Conceptualization: PL, ML-C, TP, and MT. Methodology: CP, PL, ML-C, FB, TP, and MT. Formal analysis: CP, PL, ML-C, FB, TP, and MT. Resources: MT, and PL. Writing—original draft preparation: MT and PL. Writing—review and editing: CP, PL, ML-C., FB, TP, and MT. All authors contributed to the article and approved the submitted version.

## Funding

This work was supported by Universidad de Jaén (through the program “Plan de Apoyo a la Investigación 2019–2020, Acción 1”).

## Conflict of Interest

The authors declare that the research was conducted in the absence of any commercial or financial relationships that could be construed as a potential conflict of interest.
