# Supplementary material for: Significance of PD1 Alternative Splicing in Celiac Disease as a Novel Source for Diagnostic and Therapeutic Target
Source: Front Immunol. 2021 Jun 16;12:678400. doi: 10.3389/fimmu.2021.678400 (PMC8242946; doi:10.3389/fimmu.2021.678400)
Supplement: Supplementary file 1 [file DataSheet_1.pdf]

Supplementary Material

```
                                                EXON 1
PD1_gene          ACAGTTTCCCTTCCGCTCACCTCCGCCTGAGCAGTGGAGAAGGCGGCACTCTGGTGGGGC

PD1_gene          TGCTCCAGGCATGCAGATCCCACAGGCGCCCTGGCCAGTCGTCTGGGCGGTGCTACAACT

         PD1-F primer GCGGCCAGGATGGTTCTTA ->        INTRON 1
PD1_gene          GGGCTGGCGGCCAGGATGGTTCTTAGGTAGGTGGGGTCGGCGGTCAGGTGTCCCAGAGCC
Full 687 bp             GCGGCCAGGATGGTTCTTAG
Full* 781 pb            GCGGCCAGGATGGTTCTTAG
Full* 715 bp            GCGGCCAGGATGGTTCTTAG
Δ2 327 bp               GCGGCCAGGATGGTTCTTAG
Δ3 531 bp               GCGGCCAGGATGGTTCTTAG
Δ3* 489 bp              GCGGCCAGGATGGTTCTTAG
Δ2,3 171 bp             GCGGCCAGGATGGTTCTTAG

PD1_gene          AGGGGTCTGGAGGGACCTTCCACCCTCAGTCCCTGGCAGGTCGGGGGGTGCTGAGGCGGG

PD1_gene          CCTGGCCCTGGCAGCCCAGGGGTCCCGGAGCGAGGGGTCTGGAGGGACCTTTCACTCTCA

PD1_gene          GTCCCTGGCAGGTCGGGGGGTGCTGTGGCAGGCCCAGCCTTGGCCCCCAGCTCTGCCCCT

PD1_gene          TACCCTGAGCTGTGTGGCTTTGGGCAGCTCGAACTCCTGGGTTCCTCTCTGGGCCCCAAC

PD1_gene          TCCTCCCCTGGCCCAAGTCCCCTCTTTGCTCCTGGGCAGGCAGGACCTCTGTCCCCTCTC

PD1_gene          AGCCGGTCCTTGGGGCTGCGTGTTTCTGTAGAATGACGGGTCAGGCTGGCCAGAACCCCA

PD1_gene          AACCTTGGCCGTGGGGAGTCTGCGTGGCGGCTCTGCCTTGCCCAGGCATCCTTGGTCCTC

PD1_gene          ACTCGAGTTTTCCTAAGGATGGGATGAGCCCCATGTGGGACTAACCTTGGCTTTACGACG

PD1_gene          TCAAAGTTTAGATGAGCTGGTGATATTTTTCTCATTATATCCAAAGTGTACCTGTTCGAG

PD1_gene          TGAGGACAGTTCTTCTGTCTCCAGGATCCCTCCTGGGTGGGGATTGTGCCCGCCTGGGTC

PD1_gene          TCTGCCCAGATTCCAGGGCTCTCCCCGAGCCCTGTTCAGACCATCCGTGGGGGAGGCCTT

PD1_gene          GGCCTCACTCTCCCGGATCGAGGAGAGAGGGAGCCTCTTCCTGGGCTGCCCGTGACCCTG

PD1_gene          GGCCCTCTGTGTACACTGTGACCACAGCCCGCTCCTGGACCCTCTGTGCCCGGCTGGCCC

PD1_gene          TCTGTGCCCAGCCAGCCTGCACCTGGGGATGCCAAGGCCTGGGGAGGGTGGTTTCACCCA

PD1_gene          GGCCAAGCCTAAGACAGTCCCTCTGGGCCCTGCTGGGTACCGGGGTGTGACACCACTGGG

PD1_gene          AGGACAAGATGAGGGGCACCCCTGGGGCCGCCCTGACACCCCCTCGAGGCTCCTGCCCCG

PD1_gene          GGGGTCCTGGTGCCCCTTCACTGTGGCAGGCGACTGGGGGTTCCCCACCTCGGCCCCTCT

PD1_gene          CCCGGGGCCTGCTCCCCGGCACCTGAGGCAGCATCCTTGTCAGGGCCGTGCCTTCCTGCC

PD1_gene          TCAGCGCCACCTCTTAAGGTTGGCCCGTGGGTCACTCAGGACTCAGAACTGGAGATTCTG

PD1_gene          GGCAAAAGGCAAAGAGCAAAGGGCCAAAAGGCATCCCAGGGAGACGACTGCGGGGGAACC

PD1_gene          AGAGGGCAGAGGGGCGCTCGTCACAGGGGAGGGGGAGCTGAGCGAGGCAGGAGGGGAGCC

PD1_gene          GAGCCTCTCCCCCCGTGTCCCGGCTCTTCAGGCACGCCCTCGGGACGCCACCCTCCCCGA

PD1_gene          CCCAGGCGGGAAAGATAAGAGCAAGGTGTCCGCAGCCTGACACTCGTGCCTCAGGTGCCC

PD1_gene          GCGCTTGTGCCGGACAAGACTCTCACAGGTGGCATGCCTCGGTTTCCCCACTGGTAACAG
```

PD1_gene CACAGGGCACTCAGCAAGGCGCAGTGGGCATGACTGGGGTCCTGTGGGTCCTGACCCAGA

PD1_gene TGTGGCCACCCCGGCCGCAGTGGTCTTCATTCCAGGATGCCTCTTTTCCCTCCTGATCTA

PD1_gene TTCACTGCGTTCGCCATTCGGTCATTCCCGGGGCCACCACTCCCACCTCAGGTGTGTGCT

PD1_gene TCCCTTGTGTTTTATGAGATATCCCCAACCCGGCTGCTTATTGGCCCCGTCCGAGGGCAG

PD1_gene GAGCATAAATAAGAGCCTCTGCTTTGGCGTGGGACCACTGTGAGCTCCAGTCAGCGCTGC

PD1_gene CACTGCTGAGCTCTGGGCCTTCGACAGGACTTGGCCCCTTACTGACTTCTCCGTGTGCTT

PD1_gene TGGGTCATGGGTGAGGACGCCTCCTGGCAAGGCTGCGTCCTGAGGATTAAATCGGGTCAT

PD1_gene CTGTGAAAACTACCCAGCCCAGCCCCTGACACTTTTTTGTTTGTTTCTTTTAGTGACAGG

PD1_gene GTCTTGCTCTGTCACCCAGGCTGGAGTGCAGTGGTGTGATCTCGGCTCACTCGACCTCCG

PD1_gene GGGCTCAAGCAATTCTCCCACCTCTGCCTCCAGAGTAGCTGGGACTATAGGCACGTGCCA

PD1_gene CCCTGCCAGGCTAATTTCTTCCATTTTTTTTAGAGACAGGGTCTCGCTATGTTCCCCAGG

PD1_gene CTGGGCTCAAATGGTCCTCCCACCTCAGCCTCCCCAAGTACTGGGATTACAGGCATAAGC

PD1_gene CACTGCATCTGGCCTCCATGACACATATTTTTAAAGTCTGATTTTTAAAGTCAAACTTTT

PD1_gene GAAGTCAGATTTTAAACGGACTATTTTGAAAAATATACAAAAACGTTTAAAAACAATGAA

PD1_gene TATCCCTCACCTAGAATCAATAACTAAGAATATTGACACATTTGCTTTGGGGACTGGGCG

PD1_gene GCTGGAGCTGCCATGACAAAGCTCCGCCGACCGAGTGGCTTTTAAACAGAGCCTGCCCTC

PD1_gene TCGCCGACTGAGGGCTGGACGTGCAGGATGGAGCTCCGCAGGGTCGGCTCCCCTGTGCTC

PD1_gene TGAGGGGCTCTGCTCAGCCTCTCCCGGCTGTGGCTTAAAAACAGAGCCTGTCCTCCCGCC

PD1_gene GTGGGGGGCTGGACATGCAGGACCGAGGGGCCACAGGGTCGGCTCCCTGTGCTCCGAGAG

PD1_gene GGCTCTGCTCAGCTTCTCCTGGCTGGGGGGTTTTGTGGCCACCCTCTGTGTTCCTGGGTT

PD1_gene CAGAAGCATCCCCCAGGCTCTGCCTTCATCTCTGCACGGGTGACTCTGTACAGGAAGCCA

PD1_gene GGCCTGCTGGTCAATGGCCACCCAGCCCTGTGCCCTCATCTTACCTAGTCCCAGCTGCCG

PD1_gene TCACCCTATTCCTAATAAGGCCGCCTTCTGAGGTCATGGGGTTAGGACTTCCACATAGGA

PD1_gene ATCTGTGGGGACACGGTTCGGCCCACAGCCCTTCCCACCTCCACACACACACACGACTGT

PD1_gene GAGGAGTTGGAAGACCTCACTCCTCACCCCTGCCAGGTCCTCTAGGGACAAGCTCGCTGT

PD1_gene CCTCATCCCAGCACAGCCCGTGGGACGGTTTCCTTGTCCCTAATGGGACCACGGTCAGAG

PD1_gene ATGCCGGGTCTGGTCTGGGCCAGCAGGTTCCTCCGCCCGGGGCAGGCAGCCTTCTTCTGT

PD1_gene GCGCTTCTGGAAAGCAATGTCCTGTAATGCGGTCTCTCTGCGGGAGCACCCCCACCGCCA

PD1_gene CCTCACAGGCCTGTTCCACAGCCCCGGGATGGGCTCTGTCTCCCTCCTGACCCTGCATAG

PD1_gene GGCACAGCCCTCTCTCATCAACCCACGATCCTACGTGGATCCGAGAGGGAGCACCTGGGG

PD1_gene AAACAATGGAATCCCATAGAAACACCCCAAATCTAACTTGATCCAGGACCAGCCAGTGGT

PD1_gene CACTTCTGAATATTCACCTTCCTAGTAGACACTACCAGCCAAGGGAGGCCAGGAAGCCTT

PD1_gene CCTGGAGGAGGTGGCCTGAGGACTGGGGTGAGGCAGGCCCTGCGTGGGGGTCGCCACCCA

PD1_gene GCACCCCCACACTGGGTGGGAGCCAGTCTCTGAGACTGGCTGGGGGAGGTGGGAGAGGGG

PD1_gene GCTGCTTGAACTGCAGACACCGAGGTCTAGCCCCCACCCCACCCAGCCAGTTGGTGGAGG
PD1_gene CAGGGGAGGCCGAGGGGCCCAGCTGGACCTGCTCCCCGGGGTGGATTCCAAAATAGGGGG
PD1_gene GTTGGGGGGGGCGGAACAGGAGCCCAGGGTCCTGGCTTGAGGCCCAGTGGCTGAGGGCTG
PD1_gene GTGCAAGCCAGACAGGAAAAGGGTTGAGCCTGTCAGCGCCAGCACAGATCAAGTCAGGAG
PD1_gene CAGGTCCCTCCACCAATGTGTGCAAATAAATAGCAGCTAAGTTTCCAGTTACAAGAACAA
PD1_gene TGCACAGATGGTCCCAGGGACATTGCGGTGTGGACACACAGCGGCCATTGTCCTGTCGCC
PD1_gene AGCACCTCGCCCTACAGCTGGGGGGTCCCTTAGCACTTCCTAGCCATGCAGGGTCCCTGC
PD1_gene TCACAGTACCCGTGATGACTTCTGTTCCTCACCTGCCTGTCTGTCCCGACAGCTGCATGG
PD1_gene CAGCCCTGGCCTGGGAGATGGAGACCCCGAGGGGCTGCCTGCGGTGGTGGGGCCCCTGGG
PD1_gene TCCCCACTGCATTCCCAGAAACCCAGAGGGCAGGGCATTTCCCCTGCTCTGTGCCGAGTC
PD1_gene CACCCAGCCCCAGCCTAGGCCCAGTAAGGGCTGCAGCCCACCCTGTCCCAGGCTGCCTCC
PD1_gene CAGGAGCCCTCTTGGCCCTGATGCCAGAAGCCCATCTTCCTCCATTCAGGCAGGTCTCTG
PD1_gene AGTGCCCTGGCCTGGCTGCCTGCTGGCCCTGAGAGTCACACTACCCCACAGCCCTCCTTG
PD1_gene GTCAAAATCCACTCTGGAGTGGCTGGAAGATTCCCCGGGCCCACGCCGCACACGCCTATG
PD1_gene CAGGGAGCTTCCCCTGGCCGGCCGGCAGACAAGGGCGGTCTCAGAGAGGGGGCTCACCTC
PD1_gene AGCAGCCCCTTGTGTAGCTGGCCCTCGCCCCTGCCACCTCTGGGAACACCACCAGGAAGC
PD1_gene TGGGGGACAGGCACGCAGGTGAAGGAGGCGAGCGCTTGTCAGCCGGGAGGCCATGGGCAC
PD1_gene AGAGGGAACAGGGACACCCTGGGTGGCCTCAAGGTCACTTCAAACCCCTCACTCGTCCCC
PD1_gene TGGGAGGGTGCCCAGTGAGGTTGGCACTAGGAGTTGGTCCTGGTCACATGACAGACCCAC
PD1_gene CCACCTCTGGTGTCCAGCCAGCACGCCGTGGGCCAGCCTGGCTGCAGGGACACGAGGGCA
PD1_gene GCAGCCCCCTCCTCCTCTGAGCTGGTTGCTCCTTGAGTCATCACCACCGCCTGCCACGGA
PD1_gene GGCCGCCTGTCCCAGGAAGCAGAGGGACCGCAGCTGTGGCAACCAGGGCCTGGTCTCTGT
PD1_gene GTCACCTCGCTGGGGGGCCGTGCCCAGGCCTGAGACGGAACTGAGTGACAGTGCACTGGG
PD1_gene TCTGACAGTGTGGGGCTGGCGCCATGTTTGGGGAACCCTGTGGCATGGGACCTGTGGGTG
PD1_gene AGCCGGGAAAATCACCCCGTTGCATGGCATCTCGGGCCTGGATCTTAAGCGCCTGTGTTG
PD1_gene GTGCCTCCGCCTGGCGGAAGAGCCGCGACCCCCACGTTGCCATGCGGGTATCCCAAGCCC
PD1_gene TGACCCTGGCAGGCATATGTTTCAGGAGGTCCTTGTCTTGGGAGCCCAGGGTCGGGGGCC
PD1_gene CCGTGTCTGTCCACATCCGAGTCAATGGCCCATCTCGTCTCTGAAGCATCTTTGCTGTGA
PD1_gene GCTCTAGTCCCCACTGTCTTGCTGGAAAATGTGGAGGCCCCACTGCCCACTGCCCAGGGC
PD1_gene AGCAATGCCCATACCACGTGGTCCCAGCTCCGAGCTTGTCCTGAAAAGGGGGCAAAGACT
PD1_gene GGACCCTGAGCCTGCCAAGGGGCCACACTCCTCCCAGGGCTGGGGTCTCCATGGGCAGCC
PD1_gene CCCCACCCACCCAGACCAGTTACACTCCCCTGTGCCAGAGCAGTGCAGACAGGACCAGGC
PD1_gene CAGGATGCCCAAGGGTCAGGGGCTGGGGATGGGTAGCCCCCAAACAGCCCTTTCTGGGGG
PD1_gene AACTGGCCTCAACGGGGAAGGGGGTGAAGGCTCTTAGTAGGAAATCAGGGAGACCCAAGT

```
PD1_gene        CAGAGCCAGGTGCTGTGCAGAAGCTGCAGCCTCACGTAGAAGGAAGAGGCTCTGCAGTGG

PD1_gene        AGGCCAGTGCCCATCCCCGGGTGGCAGAGGCCCCAGCAGAGACTTCTCAATGACATTCCA

PD1_gene        GCTGGGGTGGCCCTTCCAGAGCCCTTGCTGCCCGAGGGATGTGAGCAGGTGGCCGGGGAG

                                                                EXON 2
PD1_gene        GCTTTGTGGGGCCACCCAGCCCCTTCCTCACCTCTCTCCATCTCTCAGACTCCCCAGACA
Full 687 bp                                                     ACTCCCCAGACA
Full* 781 pb                                                    ACTCCCCAGACA
Full* 715 bp                                                    ACTCCCCAGACA
Δ3 531 bp                                                       ACTCCCCAGACA
Δ3* 489 bp

PD1_gene        GGCCCTGGAACCCCCCACCTTCTCCCCAGCCCTGCTCGTGGTGACCGAAGGGGACAACG
Full 687 bp     GGCCCTGGAACCCCCCACCTTCTCCCCAGCCCTGCTCGTGGTGACCGAAGGGGACAACG
Full* 781 pb    GGCCCTGGAACCCCCCACCTTCTCCCCAGCCCTGCTCGTGGTGACCGAAGGGGACAACG
Full* 715 bp    GGCCCTGGAACCCCCCACCTTCTCCCCAGCCCTGCTCGTGGTGACCGAAGGGGACAACG
Δ3 531 bp       GGGCCTGGAACCCCCCACCTTCTCCCCAGCCCTGCTCGTGGTGACCGAAGGGGACAAGG
Δ3* 489 bp                                    CCCTGCTCGTGGTGACCGAAGGGGACAACG

PD1_gene        CCACCTTCACCTGCAGCTTCTCCAACACATCGGAGAGCTTCGTGCTAAACTGGTACCGCA
Full 687 bp     CCACCTTCACCTGCAGCTTCTCCAACACATCGGAGAGCTTCGTGCTAAACTGGTACCGCA
Full* 781 pb    CCACCTTCACCTGCAGCTTCTCCAACACATCGGAGAGCTTCGTGCTAAACTGGTACCGCA
Full* 715 bp    CCACCTTCACCTGCAGCTTCTCCAACACATCGGAGAGCTTCGTGCTAAACTGGTACCGCA
Δ3 531 bp       CCACCTTCACCTGCAGCTTCTCCAACACATCGGAGAGCTTCGTGCTAAACTGGTACCGCA
Δ3* 489 bp      CCACCTTCACCTGCAGCTTCTCCAACACATCGGAGAGCTTCGTGCTAAACTGGTACCGCA

PD1_gene        TGAGCCCCAGCAACCAGACGGACAAGCTGGCCGCCTTCCCCGAGGACCGCAGCCAGCCCG
Full 687 bp     TGAGCCCCAGCAACCAGACGGACAAGCTGGCCGCCTTCCCCGAGGACCGCAGCCAGCCCG
Full* 781 pb    TGAGCCCCAGCAACCAGACGGACAAGCTGGCCGCCTTCCCCGAGGACCGCAGCCAGCCCG
Full* 715 bp    TGAGCCCCAGCAACCAGACGGACAAGCTGGCCGCCTTCCCCGAGGACCGCAGCCAGCCCG
Δ3 531 bp       TGAGCCCCAGCAACCAGACGGACAAGCTGGCCGCCTTCCCCGAGGACCGCAGCCAGCCCG
Δ3* 489 bp      TGAGCCCCAGCAACCAGACGGACAAGCTGGCCGCCTTCCCCGAGGACCGCAGCCAGCCCG
Δ2,3 171 bp

PD1_gene        GCCAGGACTGCCGCTTCCGTGTCACACAACTGCCCAACGGGCGTGACTTCCACATGAGCG
Full 687 bp     GCCAGGACTGCCGCTTCCGTGTCACACAACTGCCCAACGGGCGTGACTTCCACATGAGCG
Full* 781 pb    GCCAGGACTGCCGCTTCCGTGTCACACAACTGCCCAACGGGCGTGACTTCCACATGAGCG
Full* 715 bp    GCCAGGACTGCCGCTTCCGTGTCACACAACTGCCCAACGGGCGTGACTTCCACATGAGCG
Δ3 531 bp       GCCAGGACTGCCGCTTCCGTGTCACACAACTGCCCAACGGGCGTGACTTCCACATGAGCG
Δ3* 489 bp      GCCAGGACTGCCGCTTCCGTGTCACACAACTGCCCAACGGGCGTGACTTCCACATGAGCG

PD1_gene        TGGTCAGGGCCCGGCGCAATGACAGCGGCACCTACCTCTGTGGGGCCATCTCCCTGGCCC
Full 687 bp     TGGTCAGGGCCCGGCGCAATGACAGCGGCACCTACCTCTGTGGGGCCATCTCCCTGGCCC
Full* 781 pb    TGGTCAGGGCCCGGCGCAATGACAGCGGCACCTACCTCTGTGGGGCCATCTCCCTGGCCC
Full* 715 bp    TGGTCAGGGCCCGGCGCAATGACAGCGGCACCTACCTCTGTGGGGCCATCTCCCTGGCCC
Δ3 531 bp       TGGTCAGGGCCCGGCGCAATGACAGCGGCACCTACCTCTGTGGGGCCATCTCCCTGGCCC
Δ3* 489 bp      TGGTCAGGGCCCGGCGCAATGACAGCGGCACCTACCTCTGTGGGGCCATCTCCCTGGCCC

                                                                   INTRON 2
PD1_gene        CCAAGGCGCAGATCAAAGAGAGCCTGCGGGCAGAGCTCAGGGTGACAGGTGCGGCCTCGG
Full 687 bp     CCAAGGCGCAGATCAAAGAGAGCCTGCGGGCAGAGCTCAGGGTGACAG
Full* 781 pb    CCAAGGCGCAGATCAAAGAGAGCCTGCGGGCAGAGCTCAGGGTGACAG
Full* 715 bp    CCAAGGCGCAGATCAAAGAGAGCCTGCGGGCAGAGCTCAGGGTGACAGGTGCGGCCTCGG
Δ3 531 bp       CCAAGGCGCAGATCAAAGAGAGCCTGCGGGCAGAGCTCAGGGTGACAG
Δ3* 489 bp      CCAAGGCGCAGATCAAAGAGAGCCTGCGGGCAGAGCTCAGGGTGACAG


PD1_gene        AGGCCCCGGGGCAGGGGTGAGCTGAGCCGGTCCTGGGGTGGGTGTCCCCTCCTGCACAGG
Full* 715 bp    AGGCCCCGGGGCA

PD1_gene        ATCAGGAGCTCCAGGGTCGTAGGGCAGGGACCCCCCAGCTCCAGTCCAGGGCTCTGTCCT

PD1_gene        GCACCTGGGGAATGGTGACCGGCATCTCTGTCCTCTAGCTCTGGAAGCACCCCAGCCCCT
```

```
PD1_gene        CTAGTCTGCCCTCACCCCTGACCCTGACCCTCCACCCTGACCCCGTCCTAACCCCTGACC

                                EXON 3
PD1_gene        TTTGTGCCCTTCCAGAGAGAAGGGCAGAAGTGCCCACAGCCCACCCCAGCCCCTCACCCA
Full 687 bp                    AGAGAAGGGCAGAAGTGCCCACAGCCCACCCCAGCCCCTCACCCA
Full* 781 pb                   AGAGAAGGGCAGAAGTGCCCACAGCCCACCCCAGCCCCTCACCCA
Full* 715 bp                CAGAGAGAAGGGCAGAAGTGCCCACAGCCCACCCCAGCCCCTCACCCA
Δ2 327 bp                      AGAGAAGGGCAGAAGTGCCCACAGCCCACCCCAGCCCCTCACCCA

PD1_gene        GGCCAGCCGGCCAGTTCCAAACCCTGGTGGTTGGTGTCGTGGGCGGCCTGCTGGGCAGCC
Full 687 bp     GGCCAGCCGGCCAGTTCCAAACCCTGGTGGTTGGTGTCGTGGGCGGCCTGCTGGGCAGCC
Full* 781 pb    GGCCAGCCGGCCAGTTCCAAACCCTGGTGGTTGGTGTCGTGGGCGGCCTGCTGGGCAGCC
Full* 715 bp    GGCCAGCCGGCCAGTTCCAAACCCTGGTGGTTGGTGTCGTGGGCGGCCTGCTGGGCAGCC
Δ2 327 bp       GGCCAGCCGGCCAGTTCCAAACCCTGGTGGTTGGTGTCGTGGGCGGCCTGCTGGGCAGCC

                                                                   INTRON 3
PD1_gene        TGGTGCTGCTAGTCTGGGTCCTGGCCGTCATCTGCTCCCGGGCCGCACGAGGTAACGTCA
Full 687 bp     TGGTGCTGCTAGTCTGGGTCCTGGCCGTCATCTGCTCCCGGGCCGCACGAG
Full* 781 pb    TGGTGCTGCTAGTCTGGGTCCTGGCCGTCATCTGCTCCCGGGCCGCACGAG
Full* 715 bp    TGGTGCTGCTAGTCTGGGTCCTGGCCGTCATCTGCTCCCGGGCCGCACGAG
Δ2 327 bp       TGGTGCTGCTAGTCTGGGTCCTGGCCGTCATCTGCTCCCGGGCCGCACGAG

PD1_gene        TCCCAGCCCCTCGGCCTGCCCTGCCCTAACCCTGCTGGCGGCCCTCACTCCCGCCTCCCC

PD1_gene        TTCCTCCACCCTTCCCTCACCCCACCCCACCTCCCCCCATCTCCCCGCCAGGCTAAGTCC
Full-781-pb                                                         CTAAGTCC

PD1_gene        CTGATGAAGGCCCCTGGACTAAGACCCCCCACCTAGGAGCACGGCTCAGGGTCGGCCTGG
Full-781-pb     CTGATGAAGGCCCCTGGACTAAGACCCCCCACCTAGGAGCACGGCTCAGGGTCGGCCTGG

                                         EXON 4
PD1_gene        TGACCCCAAGTGTGTTTCTCTGCAGGGACAATAGGAGCCAGGCGCACCGGCCAGCCCCTG
Full 687 bp                              GGACAATAGGAGCCAGGCGCACCGGCCAGCCCCTG
Full* 781 pb    TGACCCCAAGTGTGTTTCTCTGCAGGGACAATAGGAGCCAGGCGCACCGGCCAGCCCCTG
Full* 715 bp                             GGACAATAGGAGCCAGGCGCACCGGCCAGCCCCTG
Δ2 327 bp                                GGACAATAGGAGCCAGGCGCACCGGCCAGCCCCTG
Δ3 531 bp                                GGACAATAGGAGCCAGGCGCACCGGCCAGCCCCTG
Δ3* 489 bp                               GGACAATAGGAGCCAGGCGCACCGGCCAGCCCCTG
Δ2,3 171 bp                              GGACAATAGGAGCCAGGCGCACCGGCCAGCCCCTG

                INTRON 4
PD1_gene        GTGAGTCTCACTCTTTTCCTGCATGATCCACTGTGCCTTCCTTCCTGGGTGGGCAGAGGT

PD1_gene        GGAAGGACAGGCTGGGACCACACGGCCTGCAGGACTCACATTCTATTATAGCCAGGACCC

PD1_gene        CACCTCCCCAGCCCCCAGGCAGCAACCTCAATCCCTAAAGCCATGATCTGGGGCCCCAGC

PD1_gene        CCACCTGCGGTCTCCGGGGGTGCCCGGCCCATGTGTGTGCCTGCCTGCGGTCTCCAGGGG

PD1_gene        TGCCTGGCCCACGCGTGTGCCCGCCTGCGGTCTCTGGGGGTGCCCGGCCCACATATGTGC

PD1_gene        CTGCCTGCGGTCTCCAGGTGTGCCCGGCCCATGCGTGTGCCCACCTGCGAGGGCGTGGGG

PD1_gene        TGGGCTTGGTCATTTCTTATCTTACATTGGAGACAGGAGAGCTTGAAAAGTCACATTTTG

PD1_gene        GAATCCTAAATCTGCAAGAATGCCAGGGACATTTCAGAGGGGGACATTGAGCCAGAGAGG

PD1_gene        AGGGGTGGTGTCCCCAGATCACACAGAGGGCAGTGGTGGGACAGCTCAGGGTAAGCAGCT

PD1_gene        CATAGTGGGGGGCCCAGGTTCGGTGCCGGTACTGCAGCCAGGCTGTGGAGCCGCGGGCCT
```

```
                                                                          EXON 5
PD1_gene        CCTTCCTGCGGTGGGCCGTGGGGCTGACTCCCTCTCCCTTTCTCCTCAAAGAAGGAGGAC
Full 687 bp                                                        AAGGAGGAC
Full* 781 pb                                                       AAGGAGGAC
Full* 715 bp                                                       AAGGAGGAC
Δ2 327 bp                                                          AAGGAGGAC
Δ3 531 bp                                                          AAGGAGGAC
Δ3* 489 bp                                                         AAGGAGGAC
Δ2,3 171 bp                                                        AAGGAGGAC

PD1_gene        CCCTCAGCCGTGCCTGTGTTCTCTGTGGACTATGGGGAGCTGGATTTCCAGTGGCGAGAG
Full 687 bp     CCCTCAGCCGTGCCTGTGTTCTCTGTGGACTATGGGGAGCTGGATTTCCAGTGGCGAGAG
Full* 781 pb    CCCTCAGCCGTGCCTGTGTTCTCTGTGGACTATGGGGAGCTGGATTTCCAGTGGCGAGAG
Full* 715 bp    CCCTCAGCCGTGCCTGTGTTCTCTGTGGACTATGGGGAGCTGGATTTCCAGTGGCGAGAG
Δ2 327 bp       CCCTCAGCCGTGCCTGTGTTCTCTGTGGACTATGGGGAGCTGGATTTCCAGTGGCGAGAG
Δ3 531 bp       CCCTCAGCCGTGCCTGTGTTCTCTGTGGACTATGGGGAGCTGGATTTCCAGTGGCGAGAG
Δ3* 489 bp      CCCTCAGCCGTGCCTGTGTTCTCTGTGGACTATGGGGAGCTGGATTTCCAGTGGCGAGAG
Δ2,3 171 bp     CCCTCAGCCGTGCCTGTGTTCTCTGTGGACTATGGGGAGCTGGATTTCCAGTGGCGAGAG

                                         <- AGGGACTCGTCTGCCTCAT PD1-R primer
PD1_gene        AAGACCCCGGAGCCCCCCGTGCCCTGTGTCCCTGAGCAGACGGAGTATGCCACCATTGTC
Full 687 bp     AAGACCCCGGAGCCCCCCGTGCCCTGTGTCCCTGAGCAGACGGAGTA
Full* 781 pb    AAGACCCCGGAGCCCCCCGTGCCCTGTGTCCCTGAGCAGACGGAGTA
Full* 715 bp    AAGACCCCGGAGCCCCCCGTGCCCTGTGTCCCTGAGCAGACGGAGTA
Δ2 327 bp       AAGACCCCGGAGCCCCCCGTGCCCTGTGTCCCTGAGCAGACGGAGTA
Δ3 531 bp       AAGACCCCGGAGCCCCCCGTGCCCTGTGTCCCTGAGCAGACGGAGTA
Δ3* 489 bp      AAGACCCCGGAGCCCCCCGTGCCCTGTGTCCCTGAGCAGACGGAGTA
Δ2,3 171 bp     AAGACCCCGGAGCCCCCCGTGCCCTGTGTCCCTGAGCAGACGGAGTA

PD1_gene        TTTCCTAGCGGAATGGGCACCTCATCCCCCGCCCGCAGGGGCTCAGCTGACGGCCCTCGG

PD1_gene        AGTGCCCAGCCACTGAGGCCTGAGGATGGACACTGCTCTTGGCCCCTCTGACCGGCTTCC

PD1_gene        TTGGCCACCAGTGTTCTGCAGACCCTCCACCATGAGCCCGGGTCAGCGCATTTCCTCAGG

PD1_gene        AGAAGCAGGCAGGGTGCAGGCCATTGCAGGCCGTCCAGGGGCTGAGCTGCCTGGGGGCGA

PD1_gene        CCGGGGCTCCAGCCTGCACCTGCACCAGGCACAGCCCCACCACAGGACTCATGTCTCAAT

PD1_gene        GCCCACAGTGAGCCCAGGCAGCAGGTGTCACCGTCCCCTACAGGGAGGGCCAGATGCAGT

PD1_gene        CACTGCTTCAGGTCCTGCCAGCACAGAGCTGCCTGCGTCCAGCTCCCTGAATCTCTGCTG

PD1_gene        CTGCTGCTGCTGCTGCTGCTGCTGCCTGCGGCCCGGGGCTGAAGGCGCCGTGGCCCTGCC

PD1_gene        TGACGCCCCGGAGCCTCCTGCCTGAACTTGGGGGCTGGTTGGAGATGGCCTTGGAGCAGC

PD1_gene        CAAGGTGCCCCTGGCAGTGGCATCCCGAAACGCCCTGGACGCAGGGCCCAAGACTGGGCA

PD1_gene        CAGGAGTGGGAGGTACATGGGGCTGGGGACTCCCCAGGAGTTATCTGCTCCCTGCAGGCC

PD1_gene        TAGAGAAGTTTCAGGGAAGGTCAGAAGAGCTCCTGGCTGTGGTGGGCAGGGCAGGAAACC

PD1_gene        CCTCCACCTTTACACATGCCCAGGCAGCACCTCAGGCCCTTTGTGGGGCAGGGAAGCTGA

PD1_gene        GGCAGTAAGCGGGCAGGCAGAGCTGGAGGCCTTTCAGGCCCAGCCAGCACTCTGGCCTCC

PD1_gene        TGCCGCCGCATTCCACCCCAGCCCCTCACACCACTCGGGAGAGGGACATCCTACGGTCCC

PD1_gene        AAGGTCAGGAGGGCAGGGCTGGGGTTGACTCAGGCCCCTCCCAGCTGTGGCCACCTGGGT

PD1_gene        GTTGGGAGGGCAGAAGTGCAGGCACCTAGGGCCCCCCATGTGCCCACCCTGGGAGCTCTC

PD1_gene        CTTGGAACCCATTCCTGAAATTATTTAAAGGGGTTGGCCGGGCTCCCACCAGGGCCTGGG

PD1_gene        TGGGAAGGTACAGGCGTTCCCCCGGGGCCTAGTACCCCCGCCGTGGCCTATCCACTCCTC
```

```
PD1_gene          ACATCCACACACTGCACCCCCACTCCTGGGGCAGGGCCACCAGCATCCAGGCGGCCAGCA

PD1_gene          GGCACCTGAGTGGCTGGGACAAGGGATCCCCCTTCCCTGTGGTTCTATTATATTATAATT

PD1_gene          ATAATTAAATATGAGAGCATGCTAAGGA
```

FIGURE S1. Alignment of the coding region of the *Homo sapiens* programmed cell death 1 (*PDCD1*) gene (accession number NC_000002.12:c241858908-241849881) with the different transcripts found in celiac patients. Primers used for RT-PCR amplification are also showed.

```
Full   687 bp   RPGWFLDSPDRPWNPPTFSPALLVVTEGDNATFTCSFSNTSESFVLNWYRMSPSNQTDKL   60
Full*  781 bp   RPGWFLDSPDRPWNPPTFSPALLVVTEGDNATFTCSFSNTSESFVLNWYRMSPSNQTDKL   60
                ************************************************************

Full   687 bp   AAFPEDRSQPGQDCRFRVTQLPNGRDFHMSVVRARRNDSGTYLCGAISLAPKAQIKESLR   120
Full*  781 bp   AAFPEDRSQPGQDCRFRVTQLPNGRDFHMSVVRARRNDSGTYLCGAISLAPKAQIKESLR   120
                ************************************************************

Full   687 bp   AELRVTERRAEVPTAHPSPSPRPAGQFQTLVVGVVGGLLGSLVLLVWVLAVICSRAARGT   180
Full*  781 bp   AELRVTERRAEVPTAHPSPSPRPAGQFQTLVVGVVGGLLGSLVLLVWVLAVICSRAARG-   179
                ***********************************************************

Full   687 bp   IGARRTGQPLKEDPSAVPVFSVDYGELDFQWREKTPEPPVPCVPEQTEX   229
Full*  781 bp   -------------------------------------------------
```

FIGURE S2. Alignment of translated sequence of the amplified region of 687 bp of the complete transcript of the *Homo sapiens* programmed cell death 1 (*PDCD1*) (accession number NM_005018) with the translated sequence of the Full 781* bp isoform found in celiacs. This isoform retain 95 bp of the intron 3. This 95 bp sequence provides a premature stop codon (TAA) that generates a truncated protein.

```
Full   687 bp   RPGWFLDSPDRPWNPPTFSPALLVVTEGDNATFTCSFSNTSESFVLNWYRMSPSNQTDKL   60
Full* 715 bp   RPGWFLDSPDRPWNPPTFSPALLVVTEGDNATFTCSFSNTSESFVLNWYRMSPSNQTDKL   60
                ************************************************************

Full   687 bp   AAFPEDRSQPGQDCRFRVTQLPNGRDFHMSVVRARRNDSGTYLCGAISLAPKAQIKESLR   120
Full* 715 bp   AAFPEDRSQPGQDCRFRVTQLPNGRDFHMSVVRARRNDSGTYLCGAISLAPKAQIKESLR   120
                ************************************************************

Full   687 bp   AELRVTERRAEVPTAHPSPSPRPAGQFQTLVVGVVGGLLGSLVLLVWVLAVICSRAARGT   180
Full* 715 bp   AELRVTGAASEAPGHREKGRSAHSPPQPLTQASRPVPNPGGWCRGRPAGQPGAASLGPGR   180
                ******    * *                          *                  *

Full   687 bp   IGARRTGQPLKEDPSAVPVFSVDYGELDFQWREKTPEPPVPCVPEQTEX          229
Full* 715 bp   HLLPGRTRDNRSQAHRPAPEGGPLSRACVLCGLWGAGFPVAREDPGAPRALCP      233
                                                      **
```

FIGURE S3. Alignment of translated sequence of the amplified region of 687 bp of the complete transcript of the *Homo sapiens* programmed cell death 1 (*PDCD1*) (accession number NM_005018) with the translated sequence of the Full 715* bp isoform found in celiacs. This isoform retain 28 bp of the intron 2. As a consequence, there is a change in the reading frame that generates a totally different sequence of amino acids.

```
Full   687 bp   RPGWFLDSPDRPWNPPTFSPALLVVTEGDNATFTCSFSNTSESFVLNWYRMSPSNQTDKL   60
Δ3*    489 bp   RPGWFL--------------ALLVVTEGDNATFTCSFSNTSESFVLNWYRMSPSNQTDKL   46
                ******              ****************************************

Full   687 bp   AAFPEDRSQPGQDCRFRVTQLPNGRDFHMSVVRARRNDSGTYLCGAISLAPKAQIKESLR   120
Δ3*    489 bp   AAFPEDRSQPGQDCRFRVTQLPNGRDFHMSVVRARRNDSGTYLCGAISLAPKAQIKESLR   106
                ************************************************************

Full   687 bp   AELRVTERRAEVPTAHPSPSPRPAGQFQTLVVGVVGGLLGSLVLLVWVLAVICSRAARGT   180
Δ3*    489 bp   AELRVT----------------------------------------------------GT   114
                ******                                                    **

Full   687 bp   IGARRTGQPLKEDPSAVPVFSVDYGELDFQWREKTPEPPVPCVPEQTEX        229
Δ3*    489-bp   IGARRTGQPLKEDPSAVPVFSVDYGELDFQWREKTPEPPVPCVPEQTET        163
                ***********************************************
```

FIGURE S4. Alignment of translated sequence of the amplified region of 687 bp of the complete transcript of the *Homo sapiens* programmed cell death 1 (*PDCD1*) (accession number NM_005018) with the translated sequence of the Δ3* isoform found in celiacs. This isoform does not show the first 42 bp of the exon 2. There is no change in the reading frame and therefore this transcript could generate a protein similar to Δ3 isoform but lacking of the first 14 amino acids formed by the exon 2.
